# Recent Insights on Catalyst Layers for Anion Exchange Membrane Fuel Cells

**DOI:** 10.1002/advs.202100284

**Published:** 2021-05-24

**Authors:** Junfeng Zhang, Weikang Zhu, Tong Huang, Chenyang Zheng, Yabiao Pei, Guoqiang Shen, Zixi Nie, Di Xiao, Yan Yin, Michael D. Guiver

**Affiliations:** ^1^ State Key Laboratory of Engines School of Mechanical Engineering Tianjin University Tianjin 300072 P. R. China; ^2^ Institute of Science and Technology China Three Gorges Corporation Beijing 100038 P. R. China

**Keywords:** anion exchange ionomer, anion exchange membrane fuel cells, catalyst layers, membrane electrode assembly, water management

## Abstract

Anion exchange membrane fuel cells (AEMFCs) performance have significantly improved in the last decade (>1 W cm^−2^), and is now comparable with that of proton exchange membrane fuel cells (PEMFCs). At high current densities, issues in the catalyst layer (CL, composed of catalyst and ionomer), like oxygen transfer, water balance, and microstructural evolution, play important roles in the performance. In addition, CLs for AEMFCs have different requirements than for PEMFCs, such as chemical/physical stability, reaction mechanism, and mass transfer, because of different conductive media and pH environment. The anion exchange ionomer (AEI), which is the soluble or dispersed analogue of the anion exchange membrane (AEM), is required for hydroxide transport in the CL and is normally handled separately with the electrocatalyst during the electrode fabrication process. The importance of the AEI–catalyst interface in maximizing the utilization of electrocatalyst and fuel/oxygen transfer process must be carefully investigated. This review briefly covers new concepts in the complex AEMFC catalyst layer, before a detailed discussion on advances in CLs based on the design of AEIs and electrocatalysts. The importance of the structure–function relationship is highlighted with the aim of directing the further development of CLs for high‐performance AEMFC.

## Graphical abstract

Because of the high pH environment in anion exchange membrane fuel cell (AEMFC), the electrochemical reaction and physical processes occurring within the catalyst layer (CL) are complex during fuel cell operation. This review introduces recent progress in materials science and microstructure control of the CL, with the aim of directing the design of high‐performance and high‐stability AEMFC.

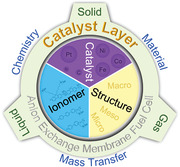

## 1 Introduction

**Scheme 1:**
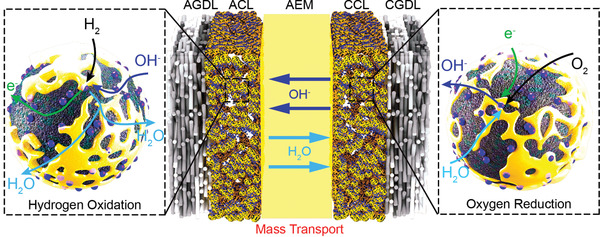
The reaction and mass transfer process of AEMFC.

As one of the core technologies of the hydrogen economy, hydrogen fuel cells have witnessed continuous improvements in material technology, such as developments in anion exchange membranes (AEMs), oxygen reduction reaction (ORR) catalysts,^[^ ^1^ ^]^ and hydrogen oxidation reaction (HOR) catalysts,^[^ ^2^, ^3^ ^]^ leading to the achievement of excellent performance in anion exchange membrane fuel cell (AEMFC). Compared with the much more established proton exchange membrane fuel cell (PEMFC), which has already been commercialized, electrochemical catalytic processes of AEMFC proceed in an alkaline environment instead of acid, thereby avoiding the strongly corrosive environment toward metallic active sites. Current AEMFCs report high maximum power densities,^[^ ^4^, ^5^ ^]^ even higher than 2 W cm^−2^.^[^ ^6^, ^7^, ^8^ ^]^ Research on AEMFC is somewhat compartmentalized, which limits a deeper understanding of the interrelationships between material science and structural properties of the catalyst layers, as shown in **Scheme** **1**. For the anode, much research has focused on exploring different materials to reduce the hydrogen binding energy (HBE) and increase the HOR activity. Since this is the region where water is generated, the anode catalyst layer (ACL) also faces problems of water management^[^ ^9^ ^]^ and hydrogen gas transfer. For the cathode, the ORR in the alkaline environment occurs more easily than in acidic environment, which allows the use of non‐platinum group metal (non‐PGM) as the ORR active center.^[^ ^10^ ^]^ In recent years, the development of nanomaterials, especially single‐atom catalysts, has provided possible application of non‐PGM catalysts in the AEMFC cathode catalyst layer (CCL).^[^ ^11^, ^12^ ^]^ However, the relationship between the catalyst and catalyst layer or membrane electrode assembly (MEA) is still unclear, but this should have a significant influence on the overall performance. In addition to the design of the catalyst and membrane/ionomer materials, the interface between them plays a dominant role in the AEMFC performance.^[^ ^13^ ^]^ As a reactant at the cathode, ambient CO_2_ in air (400 ppm) will have a detrimental impact on the AEM and AEI, which limits the deployment of AEMFC in real applications.^[^ ^14^ ^]^ Restraining the influence of CO_2_ during the fabrication and operation process is an important topic for industrial application and academic research. This review summarizes significant research of the materials used in the MEA of AEMFC. Based on material design, catalyst layer formation and the fuel cell test process are reviewed to highlight key factors for AEMFC performance. To promote the application of new materials, the mass transfer and water management of catalyst layers are emphasized, with the aim of guiding catalyst and ionomer design in the future.

## 2 Overall MEA for AEMFC

### 2.1 MEA Structure and Feature

#### 2.1.1 AEMFC Mechanism

In contrast to PEMFC, there are distinct differences in the reaction processes in both the anode and cathode catalyst layers of AEMFC. For the cathode, a surface independent outer‐sphere electron transfer component^[^ ^15^ ^]^ has been proposed to explain the fast kinetics of ORR in alkaline environments. During this process, adsorbed molecular O_2_ acts as an intermediate to accept protons from water and electrons from the electrode to accomplish the 4e^−^ reaction process in the inner‐Helmholtz plane (IHP). Compared with the inner‐sphere electron transfer process in acid, the above reaction process is more facilitative for oxygen reduction with wide‐range of non‐noble metal electrocatalysts. In the anode catalyst layer, the HOR has a greater resistance in alkaline than that in acidic environment. A study of HOR activity of Pt‐, Pd‐, and Ir‐based commercial catalysts in different pH^[^ ^16^ ^]^ indicated that the HOR activity of PGM‐based catalyst decreases significantly with the decrease of solution acidity (about 100‐fold decay for alkaline environment compared with acid). The Volmer step (M–H_ads_ + M–OH → M + H_2_O + eˉ) is shown to be the rate determining step by comparing the HOR/hydrogen evolution reaction kinetics with underpotential deposition of H (H‐UPD) charge transfer resistance values. Much research has investigated the influence of introducing oxyphilic atoms, such as Ru,^[^ ^17^ ^]^ Cu,^[^ ^18^ ^]^ and Ni^[^ ^19^ ^]^ (facilitating the adsorption of OHˉ) to vary the hydrogen binding energy,^[^ ^20^ ^]^ indicating the importance of HOR catalyst design for AEMFC applications.

#### 2.1.2 Mass Transport in AEMFC

**Figure 1:**
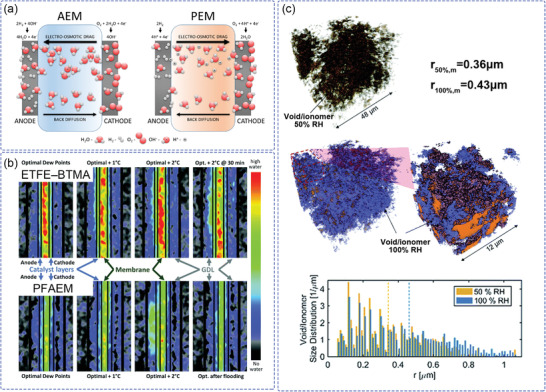
a) A comparison of water consumption, generation, migration, and diffusion in AEMFC and PEMFC. Reproduced with permission.^[^ ^21^ ^]^ Copyright 2018, Elsevier Ltd. b) Operando in‐plane neutron radiographic images of ETFE–BTMA (radiation‐modified ethylene tetrafluoroethylene with benzyltrimethylammonium (BTMA) head‐group) and perfluorinated AEMs, at 1.5 A cm^−2^ under optimized conditions. Reproduced with permission.^[^ ^24^ ^]^ Copyright 2018, The Royal Society of Chemistry. c) Volume‐rendered void/ionomer space for 50% RH and 100% RH, based on nano‐X‐ray CT characterization. The void/ionomer size distributions as well as mean radii are shown in the lower part. Reproduced with permission.^[^ ^25^ ^]^ Copyright 2018, The Royal Society of Chemistry.

With the development of non‐PGM catalysts for both the anode and cathode catalyst layers, a thorough understanding of the mass‐transfer properties of the catalyst layer, especially that with a thicker layer, should also be addressed. As the region where electrochemical reactions and mass transport occur, the MEA plays an important role in power density output. The components, morphology, and operating conditions, which relate to the transport of reactants, reaction products, ions, and water, will determine the overall performance of fuel cell. The role and distribution of water in AEMFC is different compared with that in PEMFC, as shown in **Figure** **1**a. Water molecules (generated in the anode) needs to diffuse back to the cathode and may participate in the ORR process.^[^ ^21^ ^]^ Therefore, water transport and water distribution are a major focus for AEMFC to ensure adequate hydration of the MEA without catalyst flooding.^[^ ^22^ ^]^

The transport properties of water were investigated based on a Tokuyama A201 AEM.^[^ ^23^ ^]^ The water permeability contacted by liquid water was three times higher, compared with that contacted by water vapor, indicating the important influence of the aggregation state of water. Furthermore, in‐situ AEMFC tests showed that current‐induced transport of water occurs in the opposite direction to the transport of hydroxide ions, emphasizing the larger impact of dehydration. Neutron imaging in operando has been used to allow visual water observation, to investigate water distribution in the MEA (Figure 1b).^[^ ^24^ ^]^ Based on the optimal anode and cathode operating dew points (50 °C, 62% relative humidity, RH), external water was added into the cell by increasing the dew points (1 or 2 °C increments) of both the anode and cathode sides. With the increase of dew point, water tends to accumulate in the anode gas diffusion layer (GDL), which leads to an obvious voltage decrease. The above conclusion emphasizes the critical roles of the anode, electrochemically produced water in AEM hydration and the cathode reaction, via the back‐diffusion process.

Water distribution has a significant influence on AEMFC performance, especially in anode catalyst layer. Flooding and closure of the pores in the gas diffusion electrode (GDE) limits the hydrogen mass transport, leading to poor performance and instability. More information about the catalyst layer can be obtained by micro‐ and nano‐X‐ray computed tomography (micro‐ or nano‐X‐CT).^[^ ^25^ ^]^ A comparison of the void/ionomer space at 50% or 100% RH indicates that the ionomer apparently swells from smaller to larger voids uniformly, leading to a larger pore size in the CL, which beneficially provides low resistance pathways for water removal, as shown in Figure 1c. The X‐CT results also reveal the hydrophobic nature of the nickel state in NiCu, where the NiCu surface may be isostructural *β*‐Ni(OH)_2_. The hydrophobic nature of the electrocatalyst allows for improved water distribution in the anode.

#### 2.1.3 Operating Conditions

AEMFC operating conditions, such as inlet RH, backpressure, and current density, provide external controls to lessen the impact of flooding on performance. For the anode and cathode, asymmetric pressure and RH operations were proposed to facilitate water diffusion from the anode to the cathode.^[^ ^26^ ^]^ Water vapor pressure at the cathode can be enhanced by unbalanced pressure operation, which reduces the ohmic loss and improves cell performance. On the other hand, the RH and backpressure in the anode need to be finely regulated.^[^ ^27^ ^]^ As the backpressure increases, the boiling point of water increases, which is not conducive to water removal from the anode. Fuel cell investigations underline the importance of asymmetric regulation of anode and cathode inlet gases for optimized mass transfer and electrochemical reaction environments. Similarly, the influence of RH also demonstrated that insufficient humidification of cathode is beneficial to improve fuel cell performance.^[^ ^28^ ^]^ Unsaturated humidification at the cathode can increase the water gradient between the anode and cathode, which is conducive to accelerate the back diffusion of water from anode to cathode. This not only alleviates the flooding of the anode, but also solves the problem of cathode drying.

The AEMFC catalyst layer is a complex subassembly, which is influenced by a number of different factors. A comprehensive investigation of the effects of ionomer content, inlet gas RH, anode backpressure, and role of the microporous layer (MPL) was conducted.^[^ ^29^ ^]^ It is found that better cell performance is obtained with the increase of ionomer content under various gas RH, due to the improved membrane hydration and enhanced ionic conduction in the CL. The slight increase of anode backpressure facilitates accelerated water transport from the anode to cathode and improves water management. Water balance is an important factor for fuel cell performance. On the one hand, abundant water improves the hydration of the ionomer and membrane, which leads to obtain higher ionic conductivity and lower resistance. On the other hand, excess water blocks the porous structure of CL, leading to high mass‐transfer resistance and poor electrochemical reactivity. For newly designed MEA materials (ionomer, catalyst, and AEM), the AEMFC operating conditions (RH and backpressure) need to be specifically tailored and optimized to achieve higher performance.

#### 2.1.4 CO_2_ Influence

A characteristic of AEMFCs is their susceptibility to poisoning by ambient atmospheric CO_2_, which is widely investigated to improve the performance and stability. Much work has been devoted to exploring the primary mechanisms for the voltage loss caused by CO_2_ poisoning:^[^ ^30^ ^]^ 1) slow mobility of carbonate salts, leading to high area‐specific resistance; 2) carbonates accumulate at the anode, leading to a Nernstian thermodynamic shift and anode potential increase; 3) a lower OHˉ concentration in anode leading to an increase in charge transfer resistance. As remedial measures during AEMFC operation, the presence of CO_2_‐derived bicarbonate can be reduced at high current densities^[^ ^31^ ^]^ or high operating temperature^[^ ^32^ ^]^ to obtain high electrochemical performance. However, because decarbonation of AEMFC does not occur through direct electrochemical reaction, the self‐purging process requires several hours after contamination.^[^ ^32^ ^]^ New strategies are expected to mitigate carbonate formation and the influence of CO_2_ more efficiently through developments in materials science and fuel cell operation protocols. To eliminate the influence of CO_2_ before AEMFC tests, the CL was subjected to ion exchange using KOH solution to replace the Clˉ with OHˉ.^[^ ^33^ ^]^ For MEAs with a non‐PGM CCLs, it is important to ensure sufficient ion exchange prior to the fuel cell test. This is because they tend to have relatively thicker CCLs compared with Pt/C CCLs, which leads to longer OHˉ transmission pathways and larger ion‐transfer resistance. Thus, the meticulous and integrated design of CL components and their morphological structure are essential for optimizing the fuel cell mass‐transfer and catalytic performance, compared with operating conditions optimization. Previous research on the properties of the catalyst layer provides some useful guidance on catalyst and CL design for practical high‐performance AEMFC.

#### 2.1.5 AEMFC Durability

The durability of the AEMFC is influenced by many factors, such as catalyst, ionomer, membrane, and operation conditions. Among them, the stability of the AEM and ionomer will directly determine the overall performance of the fuel cell.^[^ ^34^ ^]^ As one of the few commercial AEMs, FAA‐3 membrane from FuMA‐Tech Inc. exhibits good initial performance.^[^ ^35^ ^]^ However, the aryl ether‐containing polymer backbone of FAA‐3 is easily degraded under high pH conditions. The decomposed product reduces the local pH at the catalyst/ionomer interface and negatively affects the ORR activity and ionic conductivity, leading to poor fuel cell performance stability.^[^ ^36^ ^]^ To address this, aryl‐ether free polyaromatics for AEMFC with good stability have been developed (>2000 h; voltage decay rate: 15.36 µV h^−1^).^[^ ^37^, ^38^, ^39^ ^]^ Recently, an AEM and ionomer based on quaternized polycarbazole (QPC‐TMA) with a rigid ether‐free and curved backbone structure comprised of carbazole monomers were synthesized and applied in AEMFC.^[^ ^40^ ^]^ After immersion in 1 m KOH at 80 °C for 1000 h, the ion exchange capacity (IEC) value showed no significant difference from the initial. The above result was also confirmed by the good in‐situ stable AEMFC performance at 600 mA cm^−2^ at 60 °C for 50 h. On the other hand, the operation conditions of the AEMFC system also influence the integral durability. As is well known, the ORR in the cathode consumes H_2_O, which easily leads to local drying‐out of the cathode, especially under high current densities and low cell potential.^[^ ^41^ ^]^ Based on the above considerations, the operating conditions^[^ ^37^ ^]^ (gas flow rate, cell temperature, and relative humidity) and GDE structure^[^ ^42^ ^]^ (hydrophilicity, MPL, and GDL) must be optimized, which will be introduced in the following sections.

### 2.2 Adjustment of MEA Structure

#### 2.2.1 Influence of Polytetrafluoroethylene (PTFE)

Understanding of the mass‐transport processes is helpful for the design of MEAs. The surface hydrophobicity of the catalyst layer is one of the key parameters, which has significant influence on water transport. PTFE is a hydrophobic material commonly employed to modify the GDL surface.^[^ ^21^ ^]^ During fuel cell tests under different operation conditions, the PTFE‐treated anode GDL (5 wt%) showed higher maximum power density (MPD), compared with the GDL without PTFE. The presence of PTFE in both gas diffusion layers, combined with the high gas flow rate, resulted in the cell achieving 1.20 W cm^−2^ at 0.51 V and 2.28 A cm^−2^ with 66 mΩ cm^‐2^ (high frequency resistance). Even in full humidity, the cell can also achieve a maximum current density of 3.43 A cm^−2^ at 0.15 V.

**Figure 2:**
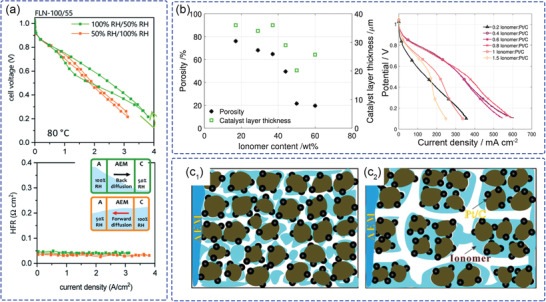
a) H_2_/O_2_ AEMFC performance of using different content ionomer electrodes for anode and cathode (anode: FLN‐100; cathode: FLN‐55). The inset figures schematically explain the hydration of the MEA component at high current density. Reproduced with permission.^[^ ^45^ ^]^ Copyright 2020, The Royal Society of Chemistry. b) Porosity and CL thickness for electrodes with various ionomer contents and corresponding polarization curves. Reproduced with permission.^[^ ^46^ ^]^ Copyright 2018, Elsevier Ltd. Schematic illustration for the CCM preparation using the c_1_) solution method and c_2_) the colloid method. Reproduced with permission.^[^ ^47^ ^]^ Copyright 2014, Elsevier Ltd.

Based on a PTFE‐treated GDL, an MPL was constructed between the catalyst layer and the GDL to improve the water management of fuel cell. The AEMFC fabricated with GDL‐30 (30 wt% PTFE) on both the anode and cathode sides exhibited a 46% improvement in the MPD, compared with that without an MPL.^[^ ^43^ ^]^ Another study evaluated the effect of MPLs to determine the characteristics of water management on both the abode and cathode.^[^ ^42^ ^]^ Compared with the cathode MPL (CMPL) case, the anode MPL (AMPL) showed the best performance under all humidity conditions, indicating that the AMPL effectively enhanced water transport from the anode to cathode across the AEM. The absence of a CMPL would further reduce the diffusion resistance of water into the CL from the flow channels. Based on observations of degradation and flooding of the GDE, a hydrophobic and stable electrode was fabricated by incorporating PTFE into the ACL. The durability tests indicated a stable microstructure without obvious catalyst agglomeration or drastic attenuation of the overall performance. However, as the inactive component in the CL, PTFE inevitably reduces the fuel cell performance, due to its large ohmic resistance and narrow pore structure. To improve the hydrophobicity and conductivity of the ACL, a new fluorine‐containing ionomer (QAPAF)^[^ ^44^ ^]^ was incorporated. As a result, the water contact angle on the ACL increased from 45° to 130° without microstructural change. The fuel cell performance could be improved further by adjusting the RH of H_2_ during fuel cell operation. Quaternized poly(fluorene) ionomers with minimal phenyl adsorption and different ion concentrations were synthesized to investigate their influence on fuel cell performance.^[^ ^45^ ^]^ Various ionomer contents in the asymmetric anode and cathode electrodes were selected and operated at different RH to investigate not only water back diffusion (anode to cathode) but also forward diffusion (cathode to anode), according to the practical environments of the anode and cathode CLs. The CCL with higher ion concentration ionomer (denoted as FLN‐100) showed forward diffusion of water even though RH is the same as ACL, which resulted in higher fuel cell performance, as shown in **Figure** **2**a.

#### 2.2.2 Control of CL Structure

To control the structure of the CL, while avoiding generating unnecessary resistances, the ratio of AEI:C:Pt was optimized to facilitate increased water capacity/tolerance, which resulted in a record for transport limiting current density (5 A cm^−2^) and maximum power density (1.9 W cm^−2^).^[^ ^21^ ^]^ Based on the Tokuyama AS‐4 ionomer, a deeper understanding^[^ ^46^ ^]^ about the performance losses was obtained by systematic investigation of the influence of ionomer content, Pt/C loading, catalyst layer thickness, RH, and anode catalyst. As shown in Figure 2b, the amount of ionomer in the CL was adjusted from 0.2 to 1.5 (AEI:Pt/C) to observe any morphology and performance changes. Excessive ionomer will reduce the porosity of the catalyst layer and block the transfer pathway for water and oxygen, leading to a large mass‐transfer resistance. Conversely, insufficient ionomer results in poor coverage and a small triple‐phase boundary (TPB), leading to the relatively lower utilization rate of active catalyst sites. Therefore, the appropriate ratio of ionomer and catalyst needs to be explored for a specific AEMFC to balance mass‐transfer resistance and TPB and achieve higher overall performance.

The CL structure and surface morphology are also determined by the catalyst ink components and the fabrication method. The solvents, which are used to disperse the catalyst and ionomer, will evaporate during CL fabrication and form porous structures. Using the same percentage of ionomer, solvents with different dielectric constant were investigated to fabricate various pore structures and TPBs of the CL.^[^ ^47^ ^]^ Catalyst ink in the colloidal state is favorable for the formation of continuous ion transport channels and large TPBs in the CL (Figure 2c), leading to higher performance in the large current density region and relatively smaller mass‐transfer resistance in the low‐frequency region.

**Figure 3:**
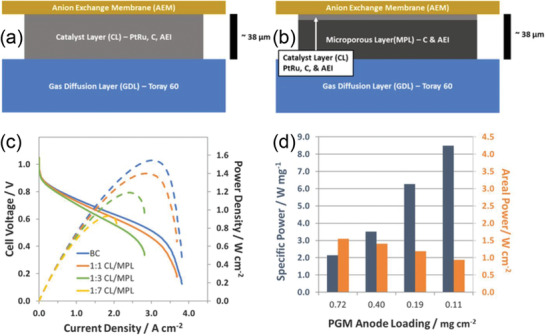
Diagram of the electrode layer designs with a) standard catalyst layer (0.72 mg_PtRu_ cm^−2^) and b) 1:7 CL/MPL multilayer electrode (0.11 mg_PtRu_ cm^−2^, thickness supplemented with MPL). c) The polarization curves of standard catalyst layer and 1:7 CL/MPL multilayer. d) A comparison of mass specific power and areal power density of AEMFCs. Reproduced under the terms of the Creative Commons Attribution 4.0 License.^[^ ^48^ ^]^ Copyright 2018, The Author(s), Published by IOP Publishing.

To reduce the usage of PGMs, a multilayered anode electrode (**Figure** **3**a,b) with optimized ratios of ionomer, carbon, and PtRu catalyst was prepared with the goal of reducing fabrication costs.^[^ ^48^ ^]^ A two‐layered anode electrode was designed to obtain a thin catalyst layer with a total PtRu loading of 0.40 mg cm^−2^ in contact with the AEM and a microporous layer in contact with the GDL had a high maximum power density of 1.4 W cm^−2^ (Figure 3c; 1:1 CL/MPL, the thickness ratio of CL and MPL is 1:1, the anode PtRu loading is 0.40 mg cm^−2^). Figure 3d shows that the specific power can be further improved to 8.5 W mg_PGM_^−1^ with a thinner CL (1:7 CL/MPL, 0.11 mg_PtRu_ cm^−2^), which meets the 2020 DOE targets (0.125 mg_PGM_ cm^−2^).^[^ ^49^ ^]^ This result highlights the important role of the MPL, which acts as a water buffer to improve the water capacity and tolerance of overall electrode. By optimizing management and more efficient use of water in the catalyst layer, a reduced content of PGM catalyst can be realized.

**Figure 4:**
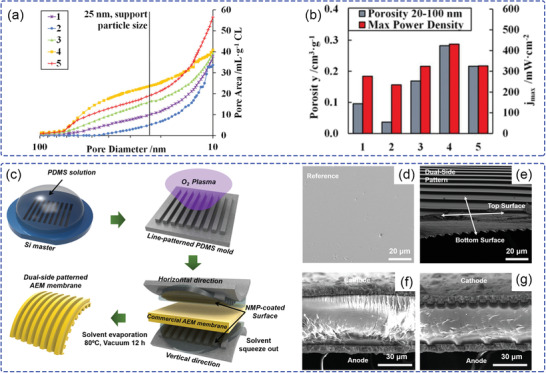
a) Cumulative pore volume curves by mercury porosimetry. b) A comparison between power densities and mesoporous internal volume. The substrate temperatures of samples 1 to 5 are 50, 80, 120, 120, and 150 °C; wait times are 30, 5, 5, 30, and 5 s, respectively. a,b) Reproduced with permission.^[^ ^35^ ^]^ Copyright 2016, The Electrochemical Society. c) Schematic of fabrication process of dual‐side patterned membrane using the solvent‐assisted molding technique. d) The SEM surface morphology of flat and dual‐side patterned membranes. Cross‐sectional SEM images of f) flat and g) dual‐side patterned MEAs. c‐f) Reproduced with permission.^[^ ^55^ ^]^ Copyright 2019, American Chemical Society.

Many AEMs have been synthesized with high ionic conductivity for AEMFC, such as poly(benzimidazolium)‐type,^[^ ^50^ ^]^ QAPS‐type polysulfone copolymer,^[^ ^51^ ^]^ interpenetrating network types^[^ ^52^ ^]^ and radiation‐grafted ETFE‐based^[^ ^53^ ^]^ membranes. However, there has been only a small level of commercialization, due to alkaline instability and large‐scale production problems. A norbornene‐based tetrablock copolymer with very high hydroxide conductivity (212 mS cm^−1^ at 80 °C)^[^ ^54^ ^]^ was synthesized for AEMFC application. Based on this AEM and ETFE‐*g*‐poly(VBTMAC) powder ionomer, with anode and cathode dew points and backpressure for the cells operating at 66 °C/75 °C, 50 kPa/100 kPa, respectively, resulted in a maximum power density of 3.5 W cm^−2^ with a maximum current density of 9.7 A cm^−2^ at 0.15 V (H_2_ and O_2_ feeds at 1 L min^−1^, 80 °C). The results were attributed to balancing and optimizing water removal and transport from the hydrogen negative electrode to the oxygen positive electrode. During the CL fabrication with FAA‐3‐Br ionomer, the boiling point of solvent *N*‐methylpyrrolidone (NMP) is very high (202 °C), resulting in a smaller pore size in the CL, compared with the CL fabricated with low boiling point solvents.^[^ ^35^ ^]^ Investigations indicate that a high proportion of micropores will improve the fuel cell performance in the kinetic‐region, but it sacrifices performance in the high current density region due to the large mass‐transfer resistance. The CL fabrication process was further optimized by adjusting the substrate temperature and wait time between depositions of successive layers of catalyst ink to obtain abundant mesopores in the CL (**Figure** **4**a) and higher power density (Figure 4b). This emphasizes the importance of CL pore control by utilizing low‐boiling solvents and constructing mesopores in the CL.

#### 2.2.3 Line‐Patterned MEA

A creative method of line‐patterned membrane/electrode interface was used to fabricate a MEA in order to optimize the interface.^[^ ^55^ ^]^ A commercially available membrane was modified by a solvent‐assisted molding technique and sandwich‐like assembly to obtain well‐aligned line arrays with a large surface area for catalyst loading, as shown in Figure 4c. Compared with the flat MEA (0.668 W cm^−2^; Figure 4d,f), both the anode‐side patterned MEA (0.841 W cm^−2^) and dual‐side patterned MEA (0.901 W cm^−2^; Figure 4e,g) exhibited better performance. The outstanding performance of the line‐patterned membrane was attributed to not only the enlarged membrane/electrode interface and microsized patterned structure, but also enhanced mass transport of the reactants, including hydroxide ions and water.

### 2.3 Half‐Cell Analysis

**Figure 5:**
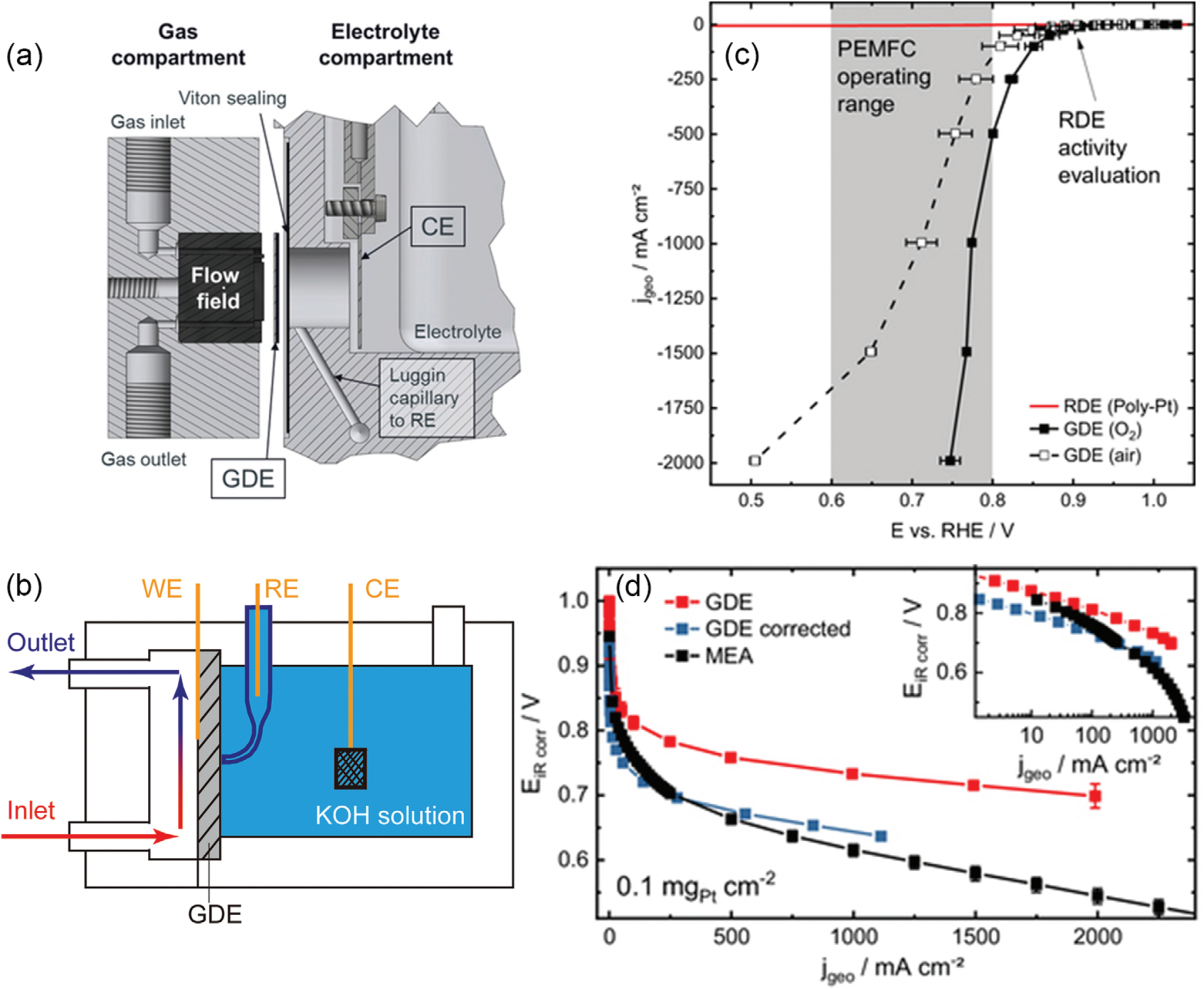
a) Detailed scheme of the GDE half‐cell setup. b) Schematic diagram of gas supply and three‐electrode connection. Comparison of half‐cell measurement with c) RDE and d) single‐cell measurements. Reproduced under the terms of the Creative Commons Attribution 4.0 License.^[^ ^56^ ^]^ Copyright 2019, The Author(s), Published by IOP Publishing.

For the application of new materials in AEMFCs, a detailed characterization of the CL is essential to understand the structure–function relationship and limiting factors. As a typical method, the rotating disk electrode (RDE) test provides a powerful tool to investigate catalytic activity in the kinetic region. However, the thin‐film catalyst layer and low‐concentration dissolved oxygen during RDE tests are limitations to a more detailed understanding of mass‐transfer processes for practical CLs in the useful voltage regions for AEMFC. The single‐cell test is more complex and is influenced by the MEA components, including the AEM, ACL as well as flow channel design, and so on. Therefore, half‐cell tests (as shown in **Figure** **5**a) were developed to evaluate CL performance with the expectation of filling the knowledge gap between RDE and MEA investigations. Compared with the established RDE method, the gas was supplied through the GDL from the gas flow channel (Figure 5b), breaking the limitation of oxygen concentration. As shown in Figure 5c,d, these can provide more information about the CL at fuel cell operation potential (0.6–0.8 V vs reversible hydrogen electrode (RHE)), while eliminating limitations of oxygen diffusion as well as the impact of AEM and anode.^[^ ^56^ ^]^ The components and structure of practical GDEs, which are influenced by the active site type and distribution of ionomer, are reflected during the ex‐situ GDE tests^[^ ^57^ ^]^ with relatively large current density. Especially in the case of AEMFC, the half‐cell method has been used to investigate the activity and stability of Fe–N–C catalyst, to simplify the test process and reveal the GDE properties.^[^ ^58^ ^]^ However, limited by the solution resistance and the reaction on counter electrode, half‐cell measurements cannot completely replace single‐cell evaluations. Therefore, multi‐scale analyses (RDE, half‐cell and single‐cell tests) are preferable to probe the key factors affecting the performance of the catalyst layer, and to gain a deeper understanding of the relationship between the catalytic activity and mass‐transfer properties.

## 3 Anode Catalyst Layer and HOR Catalyst

**Figure 6:**
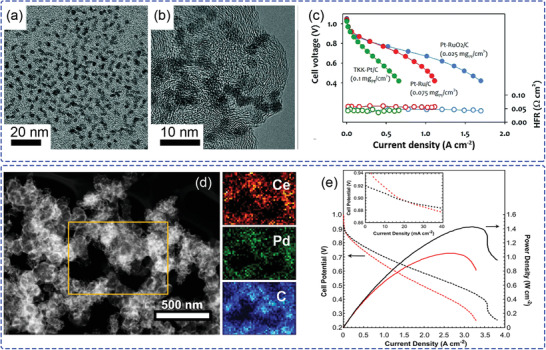
a) TEM and b) high‐resolution TEM images of PtRu nanodendrites. c) AEMFC performance comparison between MEAs with different anode catalysts (carbon supported PtRu nanodendrites catalyst, commercial Pt/C (TKK TEC10E20A, 19.4 wt% Pt), and Pt–Ru/C (Johnson Matthey HiSPEC 12100). a‐c) Reproduced with permission.^[^ ^61^ ^]^ Copyright 2020, The Royal Society of Chemistry. d) STEM‐HAADF images of Pd–CeO_2_/C and corresponding mapping of Ce (red), Pd (green), and carbon (blue) elements. e) Comparative AEMFC performance with Pd–CeO_2_/C (black curves) as the anode catalyst (0.25 mg_Pd_ cm^−2^ loading) and a reference catalyst (red curves). d,e) Reproduced with permission.^[^ ^65^ ^]^ Copyright 2019, American Chemical Society.

The HOR occurring at the AEMFC anode is one of the important operating parameters, which is much more dependent on Pt‐based catalysts compared with the ORR cathode reaction. Complicating matters, the HOR process in an alkaline environment is much more sluggish, with the exchange current density being about 100 times lower than that in an acidic environment.^[^ ^59^ ^]^ Therefore, the design strategies of catalyst and catalyst layer are under intense investigation to improve the performance of the HOR. Until now, Pt‐based catalysts are still considered to be the best option for the HOR in an alkaline environment. HOR catalysts based on Pt–Ru alloys consistently exhibit relatively higher activity, compared with those made from pure Pt, Pd, Ru, and Ir.^[^ ^48^, ^60^ ^]^ Based on detailed investigations of Pt–Ru catalysts, the Pt–RuO_2_ heterojunction is shown to be the active center for the HOR process.^[^ ^61^ ^]^ Compared with earlier alloy catalysts, ultrafine Pt nanoclusters (as shown in **Figure** **6**a,b) having unique morphology are formed. During the RDE and AEMFC tests, this Pt–RuO_2_ heterojunction catalyzes high HOR, resulting in high cell performance (Figure 6c), because of the significantly lower phenyl group adsorption properties and high accessibility of H_2_. These results provide new insights into the role of Ru, as an efficient alloy catalyst component to reduce surface phenyl group adsorption.

To reduce PGM consumption and control the surface electronic structure of Pt, a structurally ordered intermetallic alloy (O–PdFe@Pt/C) with a Pt skin on PdFe/C nanoparticles was fabricated by a structural transformation method.^[^ ^62^ ^]^ Further characterization indicated a compressively strained atomic layer and a downward shift of the *d*‐band center. Due to the influence of structurally ordered PdFe core, the HBE can be weakened, leading to very high HOR performance. Catalysts based on Pd have been widely investigated as promising candidates for the HOR in AEMFC.^[^ ^63^ ^]^ However, unmodified Pd exhibits very low HOR catalytic activity in alkaline environment (1/20 lower exchange current density than Pt/C).^[^ ^16^ ^]^ In order to increase the HOR kinetics of Pd, composites composed of Pd with CeO_2_ were explored, due to the oxygen‐deficient property of CeO_2_ having a fast saturation with OH^−^ ions and a highly oxyphilic character.^[^ ^64^ ^]^ The Pd–ceria composite interface was found to play an important role for the HOR. An organometallic cerium precursor was used to engineer the uniform Pd–CeO_2_ interfacial contact (Figure 6d) for application as an ACL in AEMFC.^[^ ^65^ ^]^ The interaction between Pd and CeO_2_ weakens the adsorption of hydrogen atoms and improves the spillover capacity of H_2_, leading to higher HOR performance. In both ex‐ and in‐situ tests, the Pd–CeO_2_/C exhibited higher current density at all potentials below 0.9 V and with significantly reduced ohmic and mass‐transfer losses, as shown in Figure 6e.

**Figure 7:**
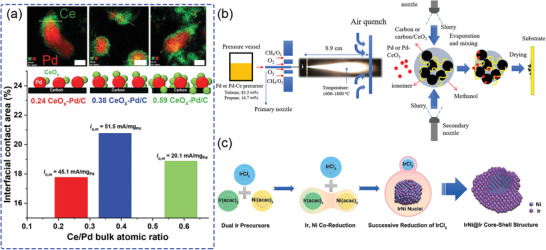
a) Interfacial contact area as a function of the Ce/Pd bulk atomic ratio. The scales in the high‐resolution STEM maps of Pd and Ce are 10, 8, and 10 nm (left to right). Reproduced with permission.^[^ ^66^ ^]^ Copyright 2020, Wiley‐VCH. b) Schematic diagram of RSDT for the synthesis of Pd–CeO_2_/C catalysts. Reproduced with permission.^[^ ^67^ ^]^ Copyright 2019, The Royal Society of Chemistry. c) Schematic diagram for the synthesis of IrNi@Ir core–shell nanoparticle. Reproduced with permission.^[^ ^68^ ^]^ Copyright 2016, The Royal Society of Chemistry.

Furthermore, the atomic ratio of Pd/Ce bulk was adjusted^[^ ^66^ ^]^ to optimize the interfacial contact area of Pd and CeO*_x_* by controlled surface reactions (CSRs). **Figure** **7**a shows the transmission electron microscopy (TEM) and electrochemical analysis have a correlation related to the interfacial contact area of Pd–CeO*_x_*. Using the ratio of 0.38 Pd–CeO*_x_*/C, the highest HOR specific exchange current density (51.5 mA mg^−1^_Pd_) was obtained from largest interfacial contact area (about 21%; Figure 7a lower pane) and a higher concentration of Pd (IV) sites, as well as an improved distribution of CeO*_x_* onto carbon supported Pd/C nanoparticles. This optimized catalyst also exhibited better performance during fuel cell single‐cell tests. The maximum power density increased from 663 mW cm^−2^ for Pd/C to 1169 mW cm^−2^ for 0.38 Pd–CeO*_x_*/C, indicating the potential of engineering the catalyst interfaces.

Flame reactive spray deposition technology (RSDT; Figure 7b) has also been utilized to enhance the interaction between Pd and CeO_2_.^[^ ^67^ ^]^ Pd and Ce precursors were sprayed from a primary nozzle and formed Pd–CeO_2_ nanoparticles on Vulcan XC‐72R carbon black, which was simultaneously sprayed from a secondary nozzle. Compared with the more established stepwise deposition of Pd and CeO_2_, this process enables homogeneous distribution and smaller particles with strong Pd–CeO_2_ interfaces, leading to the weakening of HBE, which enhances OH^−^ transfer from the anion‐conducting ionomer to the active metal surface for the HOR.

Another PGM with a similar electron structure to Pt is Ir. It has also been used to prepare Pt‐free HOR catalysts by tuning the HBE via alloying. For example, Ir–Ru alloy catalyst^[^ ^68^ ^]^ has almost four times higher HOR performance than Pt/C catalyst and is even higher than the current state‐of‐the‐art Pt–Ru/C alloy catalyst, indicating that Ir has much potential as an alloy component. However, Ir is much more expensive than Pt and has about 1/10 the natural abundance, thus requiring alloying with inexpensive metals to be of any practical use. Figure 7c illustrates the design of a carbon supported IrNi@Ir by a one‐pot synthesis for use as a HOR catalyst.^[^ ^69^ ^]^ The resulting core–shell nanoparticles, which have a synergistic effect for the HOR, were synthesized by exploiting differences in the reactivities of Ir(acac)_3_/Ni(acac)_2_ and IrCl_3_. The HBE of HOR can be finely tuned to be reduced via the electronic structure design of Ir on the surface of the alloy nanoparticle.

Perhaps the ultimate goal to reduce costs is for greater utilization of non‐PGMs and greatly reduce or even eliminate PGMs such as Pt, Pd, Ir, and Ru. Due to the high performance of Ni alloy catalysts, more Ni‐based non‐PGM HOR catalysts have been investigated. To obtain a synergistic effect for the HOR, heteroatoms, such as nitrogen, have been introduced into Ni‐based HOR catalysts with unique interface structures of Ni_3_N and Ni.^[^ ^70^ ^]^ The remarkable HOR activity of Ni_3_N/Ni on Ni foam can be attributed to 1) the unique electronic and geometrical structures for hydrogen adsorption; 2) the interface between Ni_3_N and Ni promotes water adsorption and dissociation; 3) the intrinsically metallic properties and porous morphology enable fast electron and mass transport. Based on the understanding of HOR catalytic process, carbon support with N, S, or B doped was employed to disperse the Ni nanoparticles.^[^ ^71^ ^]^ Due to the anchoring effect of S element, the S‐doped Ni‐based catalyst (Ni/SC) with smaller and highly uniform particle sizes, exhibited high specific activity (normalized by electrochemical surface area or mass). Another highly dispersed Ni_3_N/C catalyst with fine nanoparticle size (4.6 ± 1.0 nm) was synthesized on carbon support.^[^ ^72^ ^]^ Compared with bulk Ni_3_N and hybrid Ni catalysts, the mass‐normalized exchange current density of Ni_3_N/C improved significantly. As pioneering catalysts, Ketjen black‐supported Ni–Mo^[^ ^73^ ^]^ and Ni–Cu^[^ ^25^ ^]^ HOR catalysts were successively synthesized and applied in the ACL of AEMFC. The in situ performance exhibited the relatively high maximum power density (120 mW cm^−2^ for Ni–Mo and 350 mW cm^−2^ for Ni–Cu) and relative stability. However, compared with commercial Pt or Pt alloy catalyst, the lower performance of Ni‐based catalyst indicates a significant shortfall before commercial application of non‐PGM HOR catalysts can be considered practical.

## 4 Cathode Catalyst Layer and ORR Catalyst

Compared with the anode HOR, the ORR at the CCL exhibits a higher overpotential and insufficient kinetics. Until now, Pt‐based catalysts still dominate in the AEMFC cathode, due to their having the highest ORR rate in alkaline media. Similar to the ACL, high‐cost Pt‐based and other PGM catalysts are an obstacle for the development of CCL. As before, to improve the intrinsic activity of ORR for Pt‐based catalysts and reduce PGM loading, Pt‐alloy catalysts have been investigated by introducing secondary non‐PGM‐metal.^[^ ^74^ ^]^ Many low‐content‐Pt catalysts with core–shell or alloy structures, such as PtNi,^[^ ^75^ ^]^ PtCu,^[^ ^76^, ^77^ ^]^ and PtCo,^[^ ^78^, ^79^ ^]^ have been reported. To control the electronic structure and improve the intrinsic activity of non‐PGM active sites for ORR, dual‐non‐PGM active sites were introduced.^[^ ^80^ ^]^ Although the Mn–Co spinel catalyst exhibits relatively poor performance during RDE tests, it shows a higher current density in AEMFC tests, especially under low humidity conditions. The structure and surface have a synergistic effect of Mn and Co sites for binding oxygen and activating water. Different from PEMFC, H_2_O is a reactant in the ORR process for AEMFC, which requires additional consideration beyond simply tuning the reactivity of solid surfaces toward O_2_ reduction. Among non‐PGM CCLs, single‐metal atom catalysts with specific surface energy and electronic environment exhibit high ORR performance, which can be even higher than Pt/C. There also appears to be an important synergistic relationship between active metal sites and the surrounding porous structure, which can provide mass‐transfer pathways for the reaction. A Fe‐based catalyst was constructed on porous porphyrinic triazine‐based frameworks (FeSAs/PTFs) by a simple ionothermal method.^[^ ^81^ ^]^ Using spherical aberration‐corrected TEM observation and X‐ray absorption fine structure analyses, the in situ reduction of Fe element from Fe^3+^ to Fe^2+^ at high temperature was observed, which led to the Fe–N_4_ active site.

Although, many non‐PGM catalysts exhibit very high ORR performance during RDE tests, the real performance in the fuel cell is less satisfactory, due to lower mass‐specific activity, smaller pore size, and poor ionomer/catalyst distribution. Based on the development of catalyst and membrane materials, the influence of multiple conditions (anion exchange membrane, anion exchange ionomer, and electrocatalysts) were widely investigated by researchers. As shown in **Table** **1**, many non‐PGM catalysts have been developed for the ORR in alkaline environments, some of which were also applied in the CCL for AEMFC. However, the inconsistency between RDE and AEMFC performance is obvious, due to the influence of ionomer and ion exchange membrane. Among them, the most promising non‐PGM catalyst, Fe/C, exhibits very high ORR onset and half‐wave potentials during RDE tests, which is comparable or even better than Pt/C.^[^ ^82^ ^]^ But, there still remains a big performance gap between Fe/C and Pt/C catalysts during AEMFC tests, even with high non‐PGM catalyst loading (2–4 mg cm^−2^). The very low metal content and highly dispersed non‐PGM active sites combined with a high catalyst loading requirement lead to thicker catalyst layers being needed. This results in mass transport limitations of oxygen, water, and ions, as well as increases in ohmic resistances, which needs to be addressed by increasing metal content (active site density), optimizing ionomer content, and reducing the resistance of MEA.

**Table 1:** A performance comparison of different non‐PGM ORR catalysts via RDE and single‐cell tests

	RDE test				AEMFC test							
Catalyst	Ionomer	Ionomer content [wt%]	Catalyst loading [mg cm^−2^]	Half‐wave potential [V versus RHE]	Ionomer	Ionomer content [wt%]	Catalyst loading [mg cm^−2^]	Active area [cm^2^]	Anode catalyst	Membrane	Power density [mW cm^−2^]	Refs.
CNT/HDC‐1000	Nafion	10.41	0.604	0.82	I2 (Acta S.p.A.)	20–25	2	25	40 wt% Pt/C	Pore‐filled anion‐conducting membrane	221 (0.6 V)	^[^ ^93^ ^]^
g‐CN‐CNF‐700	–	–	0.501	≈0.82	AS‐4	–	2	5	40 wt% Pt/C	Not specified	171 (MPD)^a)^	^[^ ^94^ ^]^
Fe–N–CC	Nafion	75.61	0.106	0.83	AS‐4	20	0.2	5.29	40 wt% Pt/C	A201	≈120 (MPD)	^[^ ^95^ ^]^
Fe–M–LA/C‐700	Nafion	–	0.2	≈0.80	AS‐4	–	4	5	Pt/C	A201	137 (MPD)	^[^ ^96^ ^]^
CoMn/pNGr (2:1)	Nafion	27.11	0.245	0.791	FAA‐3	10	2	4	40 wt% Pt/C	FAA‐3	35 (MPD)	^[^ ^97^ ^]^
Fe/N/C	Nafion	≈10	0.4	0.93	aQAPS‐S_14_ ^b)^	20	2	4	60 wt% Pt/C	aQAPS‐S_8_	485 (MPD)	^[^ ^98^ ^]^
M/N/CDC	AS‐4	–	0.1	−0.2 versus SCE	AS‐4	≈28	1.5	4.84	46.1 wt% Pt/C	A201	80 (MPD)	^[^ ^99^ ^]^
Fe‐NMP	Nafion	12.24	0.531	0.84	AS‐4	35	3.5	5	10 wt% Pt/C	A201	218 (MPD)	^[^ ^100^ ^]^
ZIF‐CB‐700	Nafion	27.11	0.382	0.83	FAA‐3	25	2	4	60 wt% Pt/C	FAA‐3‐20	122.3 (MPD)	^[^ ^87^ ^]^
ZnCoNC‐IL20	Nafion	31.97	0.1	0.825	ETFE‐based RG‐AEI^c)^	20	2	5	PtRu/C	RG LDPE‐based benzyltrimethylammonium‐type AEM	310 (MPD)	^[^ ^101^ ^]^
MnCo_2_O_4_	Nafion	9.09	0.637	0.84	QAPPT^d)^	20	0.8	4	PtRu/C	QAPPT membrane	1200 (MPD)	^[^ ^102^ ^]^
Fe/C	Nafion	–	0.138	0.889	ETFE‐based RG‐AEI	20	1	–	Pd–CeO_2_/C	RG‐AEM	430 (0.6 V)	^[^ ^82^ ^]^
Co_3_O_4_ + C	Nafion	21.81	0.46	0.684 (onset)	aQAPS‐S_14_	20	6	13	40 wt% Pt/C	AT‐1	388 (MPD)	^[^ ^103^ ^]^
Fe–N–Gra	Nafion	31.34	0.1	0.77	HMT‐PMBI^e)^	15	2	5	PtRu/C	HMT‐PMBI	243 (MPD)	^[^ ^86^ ^]^
NiCo/NCNTs	Nafion	–	0.2	0.88	FAA‐3	≈15	4	5	Pt/C	FAA‐3‐50	65 (MPD)	^[^ ^104^ ^]^
FeSiNC_50a	Nafion	–	0.2	0.858	FAA‐3	–	3	–	60 wt% PtRu/C	FAA‐3‐50	208 (MPD)	^[^ ^12^ ^]^
Fe–N–C	Nafion	15.68	0.2	0.89	HMT‐PMBI	15	2	5	PtRu/C	HMT‐PMBI	220 (MPD)	^[^ ^105^ ^]^
FeCoNC‐at	Nafion	31.74	0.1	−0.18 versus SCE	HMT‐PMBI	≈16	2	5	Pt–Ru/C (wt% 50:25:25)	HMT‐PMBI	415 (MPD)	^[^ ^106^ ^]^
h_CoNC/CNT	Nafion	21.81	1.19	0.894	FAA‐3	–	3	5	46.5 wt% Pt/C	FAA‐3	133 (MPD)	^[^ ^107^ ^]^
Fe–N–C Act	Nafion	15	0.2	0.9	FAA‐3	–	0.25	–	60 wt% PtRu/C	FAA‐3‐50	786 (MPD)	^[^ ^108^ ^]^

^a)^ Maximum power density;

^b)^ Hydrophobic linear side chain attached to quaternary ammonium polysulfone;

^c)^ ETFE‐based radiation‐grafted (RG) anion‐exchange ionomer;

^d)^ Quaternary ammonium poly(*N*‐methylpiperidine‐*co*‐*p*‐terphenyl);

^e)^ Poly[2,2′‐(2,2″,4,4″,6,6″‐hexamethyl‐*p*‐terphenyl‐3,3″‐diyl)‐5,5′‐bi‐benzimidazole].

**Figure 8:**
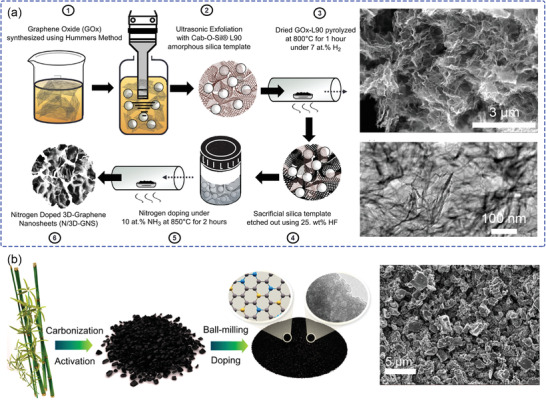
a) Synthesis of nitrogen‐doped 3D graphene nanosheets using the sacrificial support method and corresponding SEM and TEM images of the obtained catalyst. Reproduced with permission.^[^ ^91^ ^]^ Copyright 2017, American Chemical Society. b) Schematic diagram of S/N‐codoped bamboo carbon catalysts and corresponding SEM images. Reproduced with permission.^[^ ^92^ ^]^ Copyright 2019, American Chemical Society.

The hierarchical porosity and good conductivity of the carbon substrates also play an important role for achieving high AEMFC performance. Due to their electrochemical and electrically conductive properties, various carbon materials, such as carbon nanotubes (CNTs),^[^ ^83^, ^84^, ^85^ ^]^ graphene,^[^ ^86^ ^]^ carbon black,^[^ ^87^ ^]^ natural shungite,^[^ ^88^ ^]^ and carbide‐derived carbon,^[^ ^89^, ^90^ ^]^ are invariably selected as catalyst substrates. The highly porous structure of carbon materials can also provide a large surface onto which can be loaded various active components, such as M–N_4_. Using graphene carbon substrates, and employing amorphous silica as a hard template, the porosity of the CCL could be controlled, as shown in **Figure** **8**a.^[^ ^91^ ^]^ The resulting 3D nitrogen‐doped graphene supported palladium nanoparticles (Pd/3D‐GNS) were applied in AEMFC tests, which showed higher maximum power density (250 mW cm^−2^), compared with conventional carbon black supported Pd catalyst (189 mW cm^−2^). The improvement in performance can be attributed to the unique morphology and the desirable chemical composition of this support, with larger electrochemical surface area and efficient oxygen transport in the CL.

Inspired by nature, bamboo‐derived S/N‐codoped bamboo‐derived carbons (SNBCs) were developed with controlled mesopore content.^[^ ^92^ ^]^ Due to a well‐developed secondary pore structure, this catalyst exhibited good performance of ORR in alkaline environment. To adjust the microstructure of SNBC, thiourea was used to change the pore size distribution, simultaneously by the collapse and coalescence of micropores to increase the mesopores (from 20.5% for bamboo‐derived carbon to 39.9% for SNBC12 = bamboo‐derived carbon/thiourea with a ratio of 1/2). The scanning electron microscopy (SEM) images (Figure 8b) show the obvious secondary pores, which were formed between agglomerates during the fabrication of the CL, which serve as pathways for oxygen and water transport, especially at large current density.

**Figure 9:**
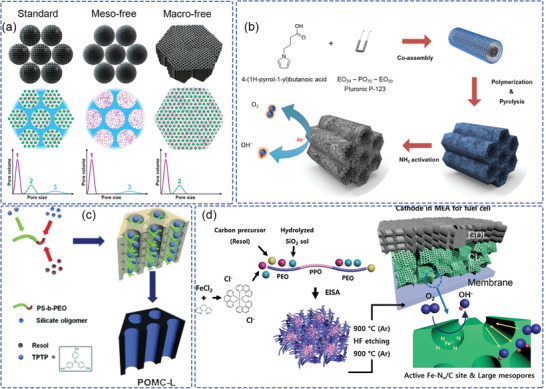
a) Illustration of three model catalysts with different porous structures. Purple, green, and blue colors indicate micro‐, meso‐, and macroporous structures, respectively. Reproduced with permission.^[^ ^109^ ^]^ Copyright 2019, American Chemical Society. b) Schematic diagram of the synthesis procedure for N‐doped hierarchical carbon catalyst. Reproduced with permission.^[^ ^110^ ^]^ Copyright 2016, The Springer Nature. c) Representation of the synthesis of POMC‐L. Reproduced with permission.^[^ ^111^ ^]^ Copyright 2015, Wiley‐VCH. d) Schematic illustration of the synthesis of m‐FePhen‐C. Reproduced with permission.^[^ ^112^ ^]^ Copyright 2018, Elsevier Ltd.

The pyrolysis process used to generate random porous structures useful for catalyst supports is difficult to control to achieve the desired pore sizes and surface compositions.^[^ ^100^ ^]^ To investigate the influence of porous structure related to catalyst function, three types of N‐doped carbon model catalysts with different ratios of micro‐, meso‐, and macropores were prepared, as shown in **Figure** **9**a.^[^ ^109^ ^]^ The electrochemically wettable area and kinetically accessible area were investigated via electrochemical impedance spectroscopy (EIS) and cyclic voltammetry (CV) methods, respectively. From physicochemical characterization and electrochemical analysis, the channel size for reaction media contributing to electrolyte wetting of the surface area is in the mesopore range, while macropores facilitate the kinetic accessibility of the active sites. Based on the understanding of porous structures present in non‐Pt catalysts, a high density of ordered N‐doped hierarchical carbon ORR catalyst (denoted as NHC) was synthesized by soft‐templating, as shown in Figure 9b.^[^ ^110^ ^]^ Due to the large accessible active surface area, the volumetric activity of NHC catalyst at 0.8 V was much higher (2600 A cm^−3^) than the 2015 DOE target (300 A cm^−3^). The ordered mesoporous structure comprising catalyst particles provides a method to fabricate porous catalyst layers that facilitates the mass transfer process for high performance MEAs.

Another nonmetal‐containing phosphorus‐doped ordered mesoporous carbon (POMC) catalyst with a controlled pore size was synthesized by block‐copolymer‐assisted evaporation‐induced self‐assembly, as shown in Figure 9c.^[^ ^111^ ^]^ It is noteworthy that the RDE and fuel cell tests have different outcomes. The POMC catalyst with microporous active sites exhibited higher performance in RDE tests than the POMC with mesoporous active sites. Conversely, the maximum power density of the POMC mesoporous catalyst was about 1.4 times higher than the POMC microporous catalyst in the fuel cell test, which indicates a large mass transfer resistance of micropores in the fuel cell for the ORR.

A number of studies have shown that transition metal‐doped catalysts exhibit outstanding ORR performance. Among them, Fe element as the active center was introduced by direct incorporation into a block copolymer assembly to obtain an ordered mesoporous Fe/N/C ORR catalyst, as shown in Figure 9d.^[^ ^112^ ^]^ The oxygen gain tests, derived from the different cell voltage between H_2_/O_2_ and H_2_/air, indicate a lower mass transfer resistance for ordered mesoporous Fe/N/C with highly active Fe–N*_x_*/C sites (denoted as m‐FePhen‐C) than for Pt/C with a similar catalyst thickness (40 µm). Another Fe‐based catalyst with smaller mesopores, with the main pore size being about 3.7 nm (denoted as sm‐FePhen‐C) was also applied in the CCL to investigate its impact to mass transfer in AEMFC. These two kinds of catalysts show almost similar kinetic activity around 0.8 V. However, the catalyst with smaller pore size exhibits higher ohmic and mass transfer losses at large current density (>400 mA cm^−2^) than m‐FePhen‐C, indicating the improved mass transfer rate of large mesopores.

**Figure 10:**
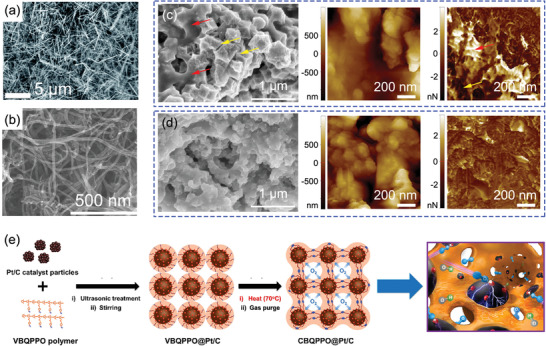
a) Surface topography of a cathode composed of unsupported Ag nanowires.^[^ ^114^ ^]^ b) SEM micrographs of CDC/CNT‐supported Co‐based catalyst. Reproduced with permission.^[^ ^117^ ^]^ Copyright 2020, American Chemical Society. SEM images and corresponding AFM topography and adhesion force mapping of c) ZIF‐derived catalyst and d) carbon black‐supported ZIF‐derived catalysts. Reproduced with permission.^[^ ^87^ ^]^ Copyright 2019, Wiley‐VCH. e) Schematic of an ionomer crosslinking immobilization strategy for CLs. Reproduced with permission.^[^ ^124^ ^]^ Copyright 2019, American Chemical Society.

A new strategy to control Zn‐ZIF‐derived ORR catalysts by changing the ratio of Zn and Co in the precursor was reported.^[^ ^113^ ^]^ During the high temperature (950 °C) activation process, the Co nanoparticles catalyze carbon rearrangement, and the evaporation of Zn metal will lead to the formation of different ratios of meso‐ and micro‐pores. The EIS data of different catalysts from RDE tests show the importance of influence of micro‐ and meso‐pores to the electrochemical surface area and mass transfer performance for ORR. A relative lower mass‐transfer resistance and higher half‐wave potential can be obtained with an optimized micro/mesopores ratio (*V*_micro_/*V*_meso_ = 0.50). In the process of catalyst deposition in the catalytic layer, mass‐transfer problems become increasingly important. To obtain higher active surface area and mass‐transfer performance, metal catalysts with unique nanomorphology have also been used for the CCL. **Figure** **10**a shows the morphology of unsupported Ag nanowires (NWs)^[^ ^114^ ^]^ prepared by a polyol method to optimize the active site and CCL structure. The Ag‐based catalyst exhibits high power density compared with state‐of‐the‐art AEMFC due to its increased electrochemical activity and elongated wire morphology with porous electrode structure, leading to high catalytic and mass‐transfer efficiency. Furthermore, the porosity and ECSA can be adjusted by the introduction of graphene nanosheet, as the support for Ag NWs, allowing a more direct oxygen diffusion pathway and larger active surface area.

CNTs, having similar relative structural dimensions to Ag NWs, also appear to be promising materials useful for catalyst synthesis, because of their large surface area, excellent electrochemical durability, high electrical conductivity, and high mechanical strength.^[^ ^115^ ^]^ Compared with carbon black, nanotube structures have a tendency to form hierarchically porous structures. A N‐doped carbide‐derived carbon/carbon nanotube (CDC/CNT)^[^ ^116^ ^]^ was prepared as the carbon substrate for Co^[^ ^117^ ^]^ and Fe^[^ ^118^, ^119^ ^]^ loading and used as the CCL for AEMFC (Figure 10b). CDC and CNT composite substrates can provide a network constructed with meso‐ and macroporous for non‐PGM‐based active site loading, leading to higher performance in the large current density region (>400 mA cm^−2^). Based on the same CDC/CNT substrate, the active site was further adjusted by using dual metal elements (Fe and Co).^[^ ^120^ ^]^ Combining the highly active site and a substrate with micro‐ and mesopores, a very high AEMFC performance (maximum power density: 1.12 W cm^−2^) was obtained. In addition, a Fe/N/C catalyst on a highly N‐doped CNT support^[^ ^98^ ^]^ was prepared by polymerization of adenosine, followed by pyrolysis. The high surface N/C atomic ratio (8%) and atomically well‐dispersed Fe on the CNT substrate provided a high accessibility of active sites for the ORR. The electronic conductivity of the CNT supported catalyst was also optimized by increasing the pyrolysis temperature from 800 to 1000 °C. The AEMFC performance using the Fe/N/C‐1000 CCL catalyst showed a higher maximum power density of 485 mW cm^−2^, indicating the key influence of catalyst electronic conductivity, which is ignored in RDE tests. Benefiting from the porous properties of CNT, it was also used as filler host substrate material to fabricate MPL, to improve the interface of membrane and CL.^[^ ^121^ ^]^ The ultralow catalyst loading CCL can be obtained by the direct deposition of Pd particles onto the surface of CNT MPL. Compared to conventional Pd/C, the maximum current density increased by 16%, indicating the importance of mass‐transfer optimization.

The ORR occurs predominately at the phase boundary, which has a dominant influence on AEMFC performance.^[^ ^122^ ^]^ Much effort has been made to optimize the TPB of the CCL for non‐PGM catalysts in AEMFC. Recently, the catalyst particle size was adjusted by incorporating nanocarbon to optimize the CCL structure and TPB area.^[^ ^87^ ^]^ Characterization by SEM and atomic force microscope (AFM) adhesion force mapping shows that the morphology and catalyst particle size have a significant influence on the distribution of ionomer, resulting in a different TPB (Figure 10c,d). Several new materials, such as layered double hydroxides (LDHs) with good conductivity have been introduced into the CCL to improve the anion transport efficiency.^[^ ^123^ ^]^ A CCL fabricated with MgAl‐LDH should improve OHˉ transport in the interlayer and surface by providing broader mass‐transfer path. However, due to its inflexibility, it is difficult to form an efficient interface between the catalyst and LDH, leading to poor fuel cell performance. To enhance the interaction between ionomer and catalyst, in situ thermal crosslinking immobilization was employed in the cathode electrode (Figure 10e).^[^ ^124^ ^]^ A more stable CL porous geometry was formed, which improved the transport pathways for oxygen and water. At the same time, the Pt/C catalyst particles were coated with intercrosslinked ionomer, forming an efficient channel for fuel permeation and ion transport. This resulted in highly efficient ion transport and large TPB, leading to high AEMFC performance (1.37 W cm^−2^).

## 5 Anion Exchange Ionomer

The AEI plays a vital role in AEM fuel cells, not simply as a binder for the catalyst/support particles and electrolyte membrane, but as a transport medium for ions, water, and gas, interacting with the catalyst responsible for the electrochemical activity. The physical and chemical stability of the AEI in the catalyst layer is an influential factor for the lifetime of the fuel cell. Thus, the importance of optimizing AEI design is increasingly realized.

### 5.1 Transport Properties

#### 5.1.1 Ion Transport

Although ion conductivity is the primary essential property of AEIs, most research has focused on ion conductivity of ionomer in the form of AEMs. According to the Nernst–Einstein relationship, ion conductivity in ionomers is related to the product of activity and effective mobility of ions.^[^ ^125^ ^]^ The activity, interpreted as ion content in the ionomer, is expressed by the IEC and degree of dissociation of ionized groups. The effective mean‐free path for conduction in ionomers is critical, as “dead‐ends” and high tortuosity lower the effective mobility of ions.^[^ ^126^ ^]^ Therefore, micromorphology is of importance to optimize topology of ionic clusters and/or conducting channels in terms of interconnectivity and tortuosity.^[^ ^127^ ^]^ Recently, advanced designs for AEMs with high ion conductivities are emerging such as those having microporous structure,^[^ ^128^ ^]^ introducing mobile ion shuttles,^[^ ^129^ ^]^ enhancing hydrogen‐bond networks,^[^ ^130^ ^]^ constructing charged 1D high‐order channels,^[^ ^131^ ^]^ decreasing the distance between conductive groups, etc.^[^ ^132^ ^]^ Theoretically, these AEM polymers have the potential to also be used as AEIs as solvent dispersions or powders. However, AEIs have specific requirements as compared with AEMs. Micrographs reveal that AEI surround the catalyst/carbon agglomerates as an ionomer skin of only a few nanometers thick, wherein the ion conductivity is distinct from bulk AEMs used in fuel cell that are usually several tens of micrometers in thickness.^[^ ^133^ ^]^ It is worth noting that the catalyst composition and its substrate affect the molecular arrangement of AEI in catalyst layers, which also influences ion conductivity of the AEI.^[^ ^134^ ^]^ Therefore, in situ electrochemical characterization is recommended to evaluate ion conductivity of AEI in catalyst layers.

#### 5.1.2 Water Transport

**Figure 11:**
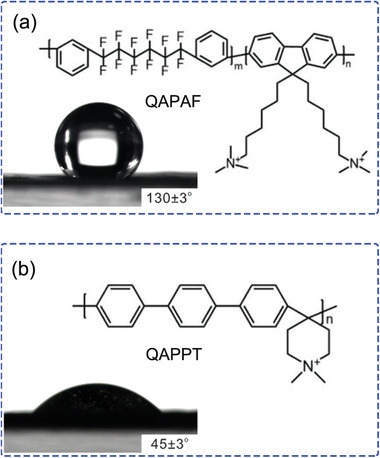
Contact angles of water droplets on the surface of the anode side of the CCM employing corresponding AEI: a) fluorine‐containing quaternary ammonia poly(arylene perfluoroalkylene) (QAPAF) and b) quaternary ammonia poly(*N*‐methyl‐piperidine‐*co*‐*p*‐terphenyl) (QAPPT). Adapted with permission.^[^ ^44^ ^]^ Copyright 2020, Elsevier Ltd.

Water transport through AEMs and AEIs is critical during AEMFC operation.^[^ ^135^ ^]^ Water serves as an essential reactant in the cathode electrochemical reaction and is a product in the anode electrochemical reaction in the cell. Flooding and drying at the ACL or CCL greatly influences AEMFC performance, especially at high current densities.^[^ ^24^ ^]^ Flooding impedes ingress of reactant gas into the CLs, while drying leads to insufficient reactant water for the cathode reaction and a substantial drop of ion conductivity of the AEI. High water permeability of the AEI is anticipated to alleviate the accumulation of water at the anode and dehydration at the cathode. This allows an increase in maximum current density by reducing mass transport limitations, while fuel cell durability is also improved because the extreme dry/wet environments can be avoided, reducing severe degradation events of cell components.^[^ ^136^ ^]^ Water diffusion normally increases with increasing free water content, and conversely, more bound water decreases water diffusion.^[^ ^137^ ^]^ Interfacial transport resistance across ionomer–vapor interfaces may contribute significantly to water transport resistance of water flux for thin ionomer films. Thus, the water content of AEI is sensitive to the operating parameters of the fuel cell.^[^ ^138^ ^]^ The hydrophobic/hydrophilic property of ionomers combined with the humidity of the flow gases can be adjusted asymmetrically to meet the particular water requirements at the anode and cathode, respectively. Kim and co‐workers adopted a highly quaternized hygroscopic ionomer (FLN‐100, IEC = 3.5 meq g^−1^) for the anode AEI. Conditions of 55% RH gas were used to restrain flooding, while the cathode AEI has a moderate IEC and 100% RH gas flow. The asymmetric electrodes enable high fuel cell performance under low RH conditions.^[^ ^45^ ^]^ Mustain and co‐workers investigated three AEIs with different IECs and optimized the corresponding RH of the feed gases. A combination of a more hydrophilic AEL (GT78) in the anode electrode and a more hydrophobic AEL (GT32) in the cathode electrode resulted in the best performance.^[^ ^37^ ^]^ **Figure** **11**a shows a fluorine‐containing hydrophobic AEI (QAPAF) reported by the group of Zhuang, in contrast to most AEIs like QAPPT in Figure 11b, which are hydrophilic. The hydrophobic nature of the AEI impedes water flooding at the anode, leading to a big gain in the cell voltage, up to 140 mV at a constant current density of 600 mA cm^−2^.^[^ ^44^ ^]^ Another fluorine‐rich ETFE‐based hydrophobic AEI reported by Varcoe group achieving high performance of fuel cell.^[^ ^139^ ^]^ To further investigate the influence of water uptake, different polymer backbones (fluorinated and hydrocarbon‐based) and diverse functional groups (various cations as part of the backbone or as pendant groups) were used to obtain the equilibrium state and kinetics parameters.^[^ ^140^ ^]^ Compared with others, poly(phenylene oxide) (PPO) AEMs functionalized with trimethylamine exhibit lower water uptake kinetics. Based on PPO AEMs, the influence of side‐chain has also been considered.^[^ ^141^ ^]^ Although a long side‐chain‐type AEM (LSCPi) shows relatively lower conductivity compared with corresponding QA‐based AEMs (LSCQA), it exhibits a higher maximum power density (116 mW cm^−2^) and better stability during AEMFC operation. In addition to the development of new AEMs, other modifications improve water transport by(1) optimizing the hydrophilic/hydrophobicity of electrodes; 2) optimizing of Pt loading and ionomer in the catalyst layer; 3) employing thin and robust AEMs.^[^ ^142^ ^]^

#### 5.1.3 Gas Transport

Different from the AEM used for AEMFC, which should minimize gas crossover, the AEI in the CL should beneficially be more permeable to oxygen and hydrogen to promote gas transport to the electrochemical reaction sites. Due to the slow diffusion rate in AEI (several nanometers thick), oxygen transport onto the Pt active sites is one of the dominant processes during fuel cell operation.^[^ ^143^, ^144^, ^145^ ^]^ Conventional polymeric ionomers usually have relatively low gas permeability, e.g., Nafion has a H_2_ permeability of only ≈7 barrer.^[^ ^36^ ^]^ Increasing the fractional free volume (FFV) of the ionomer by introducing chain‐packing disrupters like contorted spirobiindane units^[^ ^143^ ^]^ or dimethyl groups^[^ ^146^ ^]^ into the polymer backbone increases gas permeability and improves maximum power density.

### 5.2 Stability

The AEI stability is an important factor for the long‐term operational durability of fuel cells. Due to swelling and de‐swelling cycles, pressure of gases, mechanical degradation such as cracks in the CL tend to occur, leading to greater contact resistance.^[^ ^147^, ^148^ ^]^ Hydroxyl or peroxyl radicals generated by electrochemical side reactions attack vulnerable oxidizable AEI sites, leading to a decrease in molecular weight and IEC. Importantly, alkaline degradation caused by strongly nucleophilic hydroxide ions is currently the predominant degradation for ionomers in AEMFC. Hydroxide ion has been found to be more aggressive at low hydration numbers of polymer electrolyte due to loss of the protective solvation layer.^[^ ^149^ ^]^ Therefore, the cathode AEI is especially vulnerable because it operates under more dehydrating conditions resulted from water consumption of the electrochemical reaction. The easily introduced benzyl ammonium group is the most commonly employed cation for AEMFC, but it has been found to degrade in alkaline media via multiple routes. Common polymer aromatic backbones containing heteroatoms, such as polysulfone, poly(arylene ether), poly(ketone ether) etc. readily degrade by aryl–ether bond cleavage in AEIs. This decomposition pathway has been experimentally confirmed by Arges and Ramani using advanced 2D NMR techniques.^[^ ^150^ ^]^ The rapid and severe degradation of cation groups and polymer backbones lead to poor durability of AEMFC, and even affect the evaluation of maximum power output. The maximum power density of AEMFCs was typically reported as being less than 400 mW cm^−2^ before 2009.^[^ ^151^ ^]^

**Figure 12:**
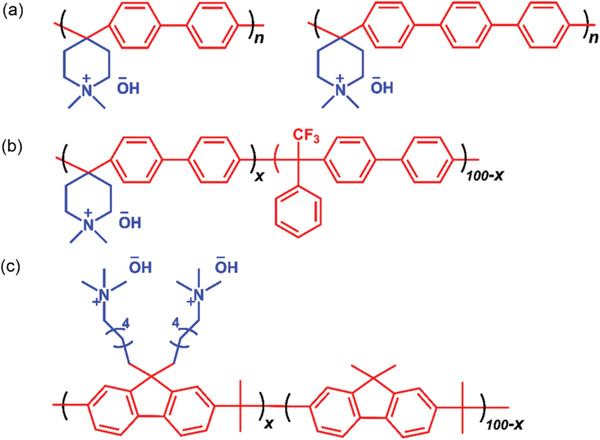
Representative alkaline stable AEI designs based on polyphenylenes for high‐performance fuel cells. a) Poly(arylene piperidinium). Reproduced with permission.^[^ ^156^ ^]^ Copyright 2018, Wiley‐VCH. b) Copolymerized poly(arylene piperidinium). Reproduced with permission.^[^ ^157^ ^]^ Copyright 2019, Springer Nature. c) Quaternized poly(fluorene). Reproduced with permission.^[^ ^159^ ^]^ Copyright 2018, Royal Society of Chemistry.

In recent years, alkaline stability of ionomers has been greatly improved owing to novel structural designs.^[^ ^104^ ^]^ Cations with good alkaline stability have been employed for the synthesis of AEIs, such as long alkyl chain ammonium, piperidinium. Other cation groups like *N*‐spirocyclic piperidinium, penta‐substituted imidazolium, cobaltocenium etc. also have good stability for AEMs, but have not been exploited as AEI in the CL. Polymers with all‐carbon backbones are proposed to withstand the alkaline operating environment of AEMFC,^[^ ^105^ ^]^ such as polyolefins and polyphenylenes. High AEMFC performances are achieved after optimization based on alkaline stable AEIs. For the polyolefin category, the Varcoe group developed ETFE‐based radiation‐grafted AEIs containing quaternary ammonium cations.^[^ ^139^ ^]^ The group of Kohl used this AEI to achieve a breakthrough of 3.4 W cm^−2^ power output with stable operation of 500 h.^[^ ^138^ ^]^ Using a more alkaline stable cation group methylpiperidinium (MPRD), better durability for fuel cell was obtained.^[^ ^152^ ^]^ Functionalized polystyrene,^[^ ^153^ ^]^ poly(vinylbenzyl chloride) (PVBC),^[^ ^154^ ^]^ and styrene‐ethylene‐butylene‐styrene (SEBS)^[^ ^155^ ^]^ are also used as AEIs due to their stable backbones. For the polyaromatic category, the group of Jannasch first developed poly(arylene piperidinium) from Friedel–Crafts type polymerization reactions of *N*‐methyl‐4‐piperidone electron‐rich phenyl monomers, as shown in **Figure** **12**a. Both backbone and cation groups are very stable and the polymer can be processed easily.^[^ ^156^ ^]^ Yan developed another poly(aryl piperidinium) AEI (Figure 12b), by introducing 2,2,2‐trifluoroacetophenone monomer to increase toughness. The MEA with an Ag‐based cathode (1 mg cm^−2^) and a low Pt anode (<150 µg cm^−2^) using the corresponding AEI and AEM exhibited a high maximum power density of 920 mW cm^−2^ at 95 °C, which had relative stable operation at a constant current density of 500 mA cm^−2^ for 300 h.^[^ ^157^ ^]^ The group of Zhuang also used the poly(aryl piperidinium) AEIs and achieved a maximum power density of AEMFC with 1.5 W cm^−2^ at 80 °C, which had stable operation over 100 h.^[^ ^158^ ^]^ The group of Kim designed an arylether‐free quaternized poly(fluorene) (FLNs) ionomer with good alkaline stability, as shown in Figure 12c. The nonrotatable fluorene groups in AEI lead to better HOR activity than rotatable phenyl group. The AEMFC exhibited a maximum power density of 1.46 W cm^−2^ at 80 °C, approaching PEMFC performance with Nafion‐based MEA.^[^ ^159^ ^]^ Although recent progress has been rapid and encouraging, there still remain challenges for improving AEMFC stability. 5000 h of stable operation at elevated temperatures and lower humidification using non‐PGM are anticipated for AEMFC.

### 5.3 Interactions with Catalyst

**Figure 13:**
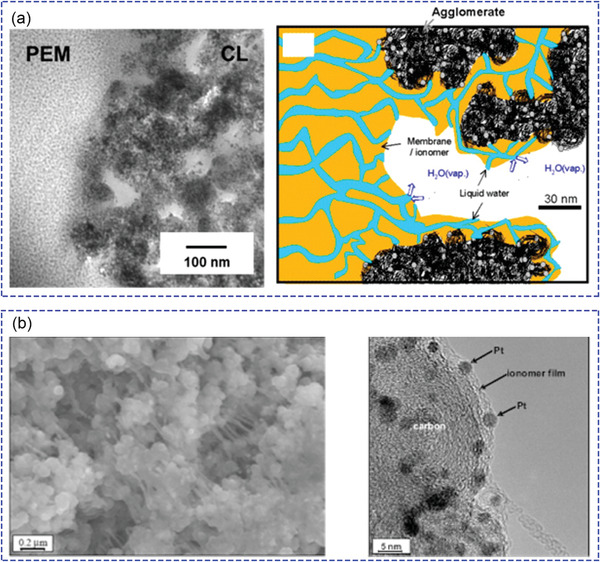
a) TEM image and simplified illustration of the membrane/catalyst layer interface. Reproduced with permission.^[^ ^160^ ^]^ Copyright 2013, American Chemical Society. b) SEM (left) and TEM images (right) showing catalyst layer and ionomer strands that bind Pt/C agglomerates, and an ultrathin ionomer film, respectively. Reproduced with permission.^[^ ^161^ ^]^ Copyright 2006, The Electrochemical Society.

Except for the development of catalysts and ionomers as individual components, multiple interactions between catalytic and AEI at each electrode must be taken into account in order to achieve optimized AEMFC performance. The interactions of AEI with catalyst/support influence electrochemical activity as well as morphologies of catalyst layer thus mass transport of electrons, ions, gas and liquid. In a typical CL, catalysts, ionomer materials and void regions form microscopic regions for electrochemical reaction, known as the TPB. Electron microscopic observations reveal the presence of aggregations of catalyst and ionomer (**Figure** **13**a).^[^ ^160^ ^]^ There is an ultrathin ionomer skin of only a few nanometers around the catalyst–carbon substrate agglomerates (Figure 13b), indicating close interactions between ionomer and catalyst in idealized TPBs.^[^ ^161^ ^]^ At present, the TPB for PEMFC is relatively more investigated, while the catalyst AEI interaction and corresponding TPB of AEMFC is less understood.

**Figure 14:**
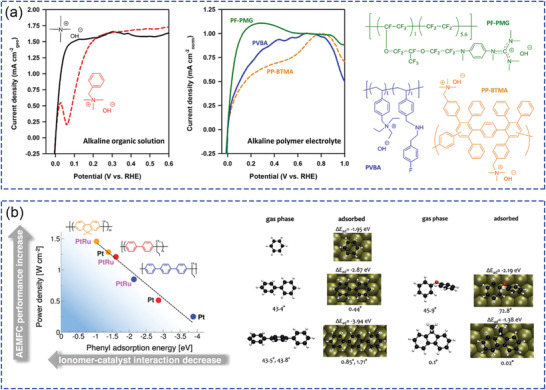
a) HOR voltammograms of Pt/C catalysts in 0.1 m TMAOH and phenyl containing BTMAOH organic solution; HOR voltammograms of Pt interacted with three AEIs containing different phenyl group amounts. Reproduced with permission.^[^ ^166^ ^]^ Copyright 2018, American Chemical Society. b) Relationship of AEMFC performances of different AEI structures and their phenyl adsorption energies; DFT‐optimized geometries of various aromatic compounds and calculated adsorption energies. Reproduced with permission.^[^ ^167^ ^]^ Copyright 2019, American Chemical Society.

The interactions between ionomer and catalyst can be characterized by adsorption energy. The AEI is known to affect the kinetics of the ORR and HOR mainly by two mechanisms: (a) cation‐hydroxide–water coadsorption, which inhibits the HOR reaction by limiting hydrogen diffusion through the coadsorbed trilayer, and (b) phenyl group adsorption leading to strong interactions and hybridization of the aromatic *π*‐orbitals with metal electronic states. Cation–hydroxide‐associated water can be adsorbed at catalyst surfaces such as Pt, which adversely impacts the HOR activity. The coadsorption may block some of the active sites of catalyst and increase the barrier for hydrogen access.^[^ ^162^, ^163^, ^164^, ^165^ ^]^ The group of Kim observed that phenyl groups often present in polyaromatic AEI reduce the HOR activity leading to inferior AEMFC performance. As is shown in **Figure** **14**a, Pt/C catalyst in phenyl containing benzyltrimethylammonium hydroxide (BTMAOH) organic solution exhibited a clear drop of HOR current density during 0.0 to 0.06 V, compared with phenyl free tetramethylammonium hydroxide (TMAOH) solution. Similarly, AEIs with higher phenyl contents result in inferior HOR activity of Pt in microelectrode minicells.^[^ ^166^ ^]^ Density functional theory (DFT) calculations on a Pt catalyst surface revealed adsorption energy for phenyl groups with different structures, which can direct the reasonable design of AEI and catalyst for AEMFC, as shown in Figure 14b.^[^ ^167^ ^]^

Both cation–hydroxide–water coadsorption and phenyl adsorption are necessary to be mitigated by comprehensive design of AEI and catalyst. The group of Dekel reported Pd‐based catalyst instead of Pt‐based catalyst to alleviate cation–hydroxide–water coadsorption at the catalyst surface, due to its strong hydrogen absorption characteristic. However, Pd‐based catalyst is more susceptible to phenyl adsorption and hydrogenation. This negative effect was minimized by using a poly(fluorene) AEI with low phenyl adsorption, the optimized design of catalyst and AEI generated improved AEMFC performance and short‐term stability.^[^ ^168^ ^]^

**Figure 15:**
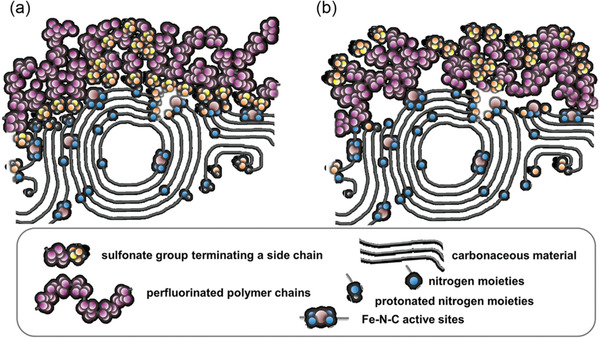
Two types of morphologies of ionomer layers caused by different catalyst–ionomer interactions, resulting in a) abundant sulfonate groups facing the pore and b) limited sulfonate groups. Reproduced with permission.^[^ ^172^ ^]^ Copyright 2017, American Chemical Society.

Ionomer–catalyst interactions also influence the structure and morphology of the CL, and thus the transport processes of electrons, ions, reactant gas, and water for the electrochemical reactions. Several molecular dynamics (MD) revealed that ionomer tends to form a thin layer on the surface of catalyst due to specific interactions.^[^ ^169^, ^170^ ^]^ The group of Jang^[^ ^171^ ^]^ calculated the binding energies of Nafion with Pt nanoparticles using quantum mechanical DFT and molecular dynamics. It is inferred that sulfonate groups tend to be chemisorbed while CF_3_CF_3_ and CF_3_OCF_3_ tend to be physisorbed. Artyushkova et al.^[^ ^172^ ^]^ adjusted the surface chemistry of transition metal–nitrogen–carbon electrocatalysts instead of noble Pt/C catalyst and calculated the interactions with ionomer by XPS and DFT. It was found that different interactions between catalyst and ionomer affect the micromorphology of ionomers layers and thus the morphology of the TPB. Two extreme cases are shown in **Figure** **15**. If the catalyst has small amounts of N*_x_*–M centers, surface carbon oxides, and cationic nitrogen, then the hydrophilic side chain of ionomer tends to face the pores, potentially causing flooding and blockage of reactive sites. Conversely, an excess of the same N*_x_*–M centers at the surface would cause strong interactions with the side chains, which would increase the barrier for ion and water transport due to more hydrophobic pores.

### 5.4 New AEI Design

**Figure 16:**
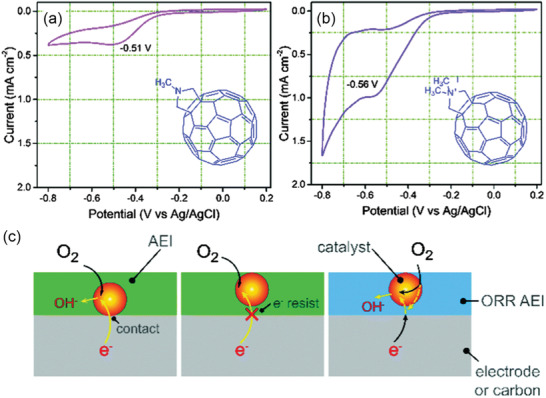
Cyclic voltammetric curves of a) MPC60 and b) quaternized *N*‐methyl pyrrolidine‐C60 (QMPC60) AEI in O_2_ saturated 0.1 m KOH, showing certain ORR activity. c) Schematic illustration of ionomer–catalyst interactions when they are in direct contact, surrounded by common AEI, and when AEI with ORR activity is employed. Reproduced with permission.^[^ ^173^ ^]^ Copyright 2016, Royal Society of Chemistry.

In a novel approach, the group of Xu prepared an AEI with fullerene attached to the cationic head groups, as shown in **Figure** **16**, the fullerene molecule imparts some ORR activity to the AEI. Thus, when catalyst particles are completely enveloped by AEI, the intrinsic ORR ability decreases resistance, reducing poisoning of conventional cationic head groups on the ORR catalyst.^[^ ^173^ ^]^ The group of Xu developed a composite material by immobilizing molecular catalyst tetrakis(4‐methoxyphenyl)‐porphyrin cobalt(II) (TMPPCo) on the side chains of an ionomer (polyfluorene, PF) homogeneous catalysis layer by immobilizing a molecular catalyst TMPPCo on the side chains of an ionomer (polyfluorene, PF). This strategy of forming homogeneous catalyst environment can lead to greatly improved utilization of the catalyst molecules.^[^ ^174^ ^]^

The group of Xu also developed an immobilization method of Pt catalyst nanoparticles by ionomer crosslinking. Following thermally initiated crosslinking of terminal vinyl groups in the AEI, an interconnected ionomer network forms ion‐conductive shells around Pt/C aggregates, thus largely preventing nanoparticle coalescence. A cell performance of up to 1.37 W cm^−2^ at 70 °C, 0.1 MPa back‐pressurization was obtained.^[^ ^124^ ^]^

## 6 Summary and Perspective

Due to the high pH environment in AEMFC compared with PEMFC, not only the reaction mechanism (HOR, ORR) proceeds by different pathways, but also the water/ion transport is opposite. Thanks to advanced molecular design of AEMs or AEIs, the AEMFC performance has been greatly improved in the last decade, but the current knowledge on the MEA/CL mass transfer properties is insufficient.

1) Most MEA or mass transfer studies are still based on commercial FAA‐3 (FuMA‐Tech), A901 or A201 (Tokuyama) AEM and AEI (e.g., FAA‐3SOLUT‐10, AS‐4), which result in AEMFC performance of around 200–500 mW cm^−2^, far below the recently reported high AEMFC power densities of >2 W cm^−2^. Thus, a deeper and more thorough understanding of the mass transfer properties within the MEA at high operating current is desired.
2) Water removal from the anode, where it is generated, is important for AEMFC performance. At present, applying asymmetric reactant conditions (e.g., pressure, RH, and temperature) has been proved to be effective, while the adjustment of MEA or CL component is considered to be the promising pathway for improving the water management in the anode.
3) Although there are many studies reporting individual non‐PGM ORR catalyst systems, which show high activity based on RDE tests in alkaline, translating this into high performance AEMFC is much more elusive, because it is a multicomponent and multiscale integrated device involving mass activity and mass transfer at the nanoscale. Thus, the evaluation of catalysts by RDE cannot properly reflect actual application in fuel cell. Typically, non‐PGM catalysts have higher specific surface area (porosity) and relatively larger particle size comparing with commercial Pt/C. Further investigations on non‐PGM CLs are of importance to achieving cost reductions.
4) The TPB interface between the catalyst and ionomer is the primary region where electrochemical reaction occurs, but the mechanisms involved are not fully understood. Some advanced ionomer structures are not given. A better understanding of the interactions between the AEI and non‐PGM active sites is needed.
5) Knowledge gained from PEMFCs shows that the MEA fabrication process dominates the final CL microstructure. The influence of atmospheric CO_2_ on the AEM and AEI during MEA process needs deeper investigation.

## Conflict of Interest

The authors declare no conflict of interest.

## References

[1] X. Ge , A. Sumboja , D. Wuu , T. An , B. Li , F. T. Goh , T. A. Hor , Y. Zong , Z. Liu , ACS Catal. 2015, 5, 4643.

[2] C. A. Campos‐Roldán , N. Alonso‐Vante , Electrochem. Energy Rev. 2019, 2, 312.

[3] E. S. Davydova , S. Mukerjee , F. Jaouen , D. R. Dekel , ACS Catal. 2018, 8, 6665.

[4] X. Peng , T. J. Omasta , E. Magliocca , L. Wang , J. R. Varcoe , W. E. Mustain , Angew. Chem. 2019, 131, 1058. 10.1002/anie.201811099 PMC6468319 30414220

[5] L. Wang , J. J. Brink , J. R. Varcoe , Chem. Commun. 2017, 53, 11771. 10.1039/c7cc06392j 29034381

[6] L. Wang , M. Bellini , H. A. Miller , J. R. Varcoe , J. Mater. Chem. A 2018, 6, 15404.

[7] G. Huang , M. Mandal , X. Peng , A. C. Yang‐Neyerlin , B. S. Pivovar , W. E. Mustain , P. A. Kohl , J. Electrochem. Soc. 2019, 166, F637.

[8] L. Wang , X. Peng , W. E. Mustain , J. R. Varcoe , Energy Environ. Sci. 2019, 12, 1575.

[9] J. Zhang , Y. Liu , W. Zhu , Y. Pei , Y. Yin , Y. Qin , X. Li , M. D. Guiver , Cell Rep. Phys. Sci. 2021, 2, 100377.

[10] S. Fu , C. Zhu , J. Song , D. Du , Y. Lin , Adv. Energy Mater. 2017, 7, 1700363.

[11] A. Sarapuu , E. Kibena‐Põldsepp , M. Borghei , K. Tammeveski , J. Mater. Chem. A 2018, 6, 776.

[12] H. S. Kim , C. H. Lee , J.‐H. Jang , M. S. Kang , H. Jin , K.‐S. Lee , S. U. Lee , S. J. Yoo , W. C. Yoo , J. Mater. Chem. A 2021, 9, 4297.

[13] W. E. Mustain , M. Chatenet , M. Page , Y. S. Kim , Energy Environ. Sci. 2020, 13, 2805.

[14] T. Reshetenko , M. Odgaard , D. Schlueter , A. Serov , J. Power Sources 2018, 375, 185.

[15] N. Ramaswamy , S. Mukerjee , J. Phys. Chem. C 2011, 115, 18015.

[16] J. Durst , A. Siebel , C. Simon , F. Hasche , J. Herranz , H. Gasteiger , Energy Environ. Sci. 2014, 7, 2255.

[17] L. L. Mickelson , C. Friesen , J. Am. Chem. Soc. 2009, 131, 14879. 19778065 10.1021/ja904432c

[18] S. M. Alia , B. S. Pivovar , Y. Yan , J. Am. Chem. Soc. 2013, 135, 13473. 23952885 10.1021/ja405598a

[19] M. Alesker , M. Page , M. Shviro , Y. Paska , G. Gershinsky , D. R. Dekel , D. Zitoun , J. Power Sources 2016, 304, 332.

[20] J. Zheng , W. Sheng , Z. Zhuang , B. Xu , Y. Yan , Sci. Adv. 2016, 2, e1501602. 27034988 10.1126/sciadv.1501602 PMC4803484

[21] T. J. Omasta , L. Wang , X. Peng , C. A. Lewis , J. R. Varcoe , W. E. Mustain , J. Power Sources 2018, 375, 205.

[22] T. D. Myles , A. M. Kiss , K. N. Grew , A. A. Peracchio , G. J. Nelson , W. K. Chiu , J. Electrochem. Soc. 2011, 158, B790.

[23] B. Eriksson , H. Grimler , A. Carlson , H. Ekström , R. W. Lindström , G. Lindbergh , C. Lagergren , Int. J. Hydrogen Energy 2019, 44, 4930.

[24] T. J. Omasta , A. M. Park , J. M. LaManna , Y. Zhang , X. Peng , L. Wang , D. L. Jacobson , J. R. Varcoe , D. S. Hussey , B. S. Pivovar , W. E. Mustain , Energy Environ. Sci. 2018, 11, 551.

[25] A. Roy , M. R. Talarposhti , S. J. Normile , I. V. Zenyuk , V. De Andrade , K. Artyushkova , A. Serov , P. Atanassov , Sustainable Energy Fuels 2018, 2, 2268.

[26] D. Saebea , C. Chaiburi , S. Authayanun , Chem. Eng. J. 2019, 374, 721.

[27] T. Wang , L. Shi , J. Wang , Y. Zhao , B. P. Setzler , S. Rojas‐Carbonell , Y. Yan , J. Electrochem. Soc. 2019, 166, F3305.

[28] X. Gao , H. Yu , B. Qin , J. Jia , J. Hao , F. Xie , Z. Shao , Polym. Chem. 2019, 10, 1894.

[29] H. Deng , D. Wang , R. Wang , X. Xie , Y. Yin , Q. Du , K. Jiao , Appl. Energy 2016, 183, 1272.

[30] Y. Zheng , G. Huang , L. Wang , J. R. Varcoe , P. A. Kohl , W. E. Mustain , J. Power Sources 2020, 467, 228350.

[31] H.‐S. Shiau , I. V. Zenyuk , A. Z. Weber , J. Electrochem. Soc. 2017, 164, E3583.

[32] Y. Zheng , T. J. Omasta , X. Peng , L. Wang , J. R. Varcoe , B. S. Pivovar , W. E. Mustain , Energy Environ. Sci. 2019, 12, 2806.

[33] M. J. Kim , O. H. Kim , S. Kim , Y. W. Choi , Y. H. Cho , Y. E. Sung , J. Ind. Eng. Chem. 2018, 61, 437.

[34] X. Gao , H. Yu , J. Jia , J. Hao , F. Xie , J. Chi , B. Qin , L. Fu , W. Song , Z. Shao , RSC Adv. 2017, 7, 19153.

[35] B. Britton , S. Holdcroft , J. Electrochem. Soc. 2016, 163, F353.

[36] E. J. Park , S. Maurya , A. S. Lee , D. P. Leonard , D. Li , J. Y. Jeon , C. Bae , Y. S. Kim , J. Mater. Chem. A 2019, 7, 25040.

[37] N. Ul Hassan , M. Mandal , G. Huang , H. A. Firouzjaie , P. A. Kohl , W. E. Mustain , Adv. Energy Mater. 2020, 10, 2001986.

[38] K. Yang , X. Chu , X. Zhang , X. Li , J. Zheng , S. Li , N. Li , T. A. Sherazi , S. Zhang , J. Membr. Sci. 2020, 603, 118025.

[39] F. Zhang , T. Li , W. Chen , X. Wu , X. Yan , W. Xiao , Y. Zhang , X. Wang , G. He , J. Membr. Sci. 2021, 624, 119052.

[40] M. S. Cha , J. E. Park , S. Kim , S.‐H. Han , S.‐H. Shin , S. H. Yang , T.‐H. Kim , D. M. Yu , S. So , Y. T. Hong , S. J. Yoon , S.‐G. Oh , S. Y. Kang , O.‐H. Kim , H. S. Park , B. Bae , Y.‐E. Sung , Y.‐H. Cho , J. Y. Lee , Energy Environ. Sci. 2020, 13, 3633.

[41] P. Veh , B. Britton , S. Holdcroft , R. Zengerle , S. Vierrath , M. Breitwieser , RSC Adv. 2020, 10, 8645. 10.1039/c9ra09628k PMC9050023 35496547

[42] X. Xie , J. Zhou , S. Wu , J. W. Park , K. Jiao , Int. J. Energy Res. 2019, 43, 8522.

[43] V. M. Truong , C.‐L. Wang , M. Yang , H. Yang , J. Power Sources 2018, 402, 301.

[44] M. Hu , Q. Li , H. Peng , H. Ma , L. Xiao , G. Wang , J. Lu , L. Zhuang , J. Power Sources 2020, 472, 228471.

[45] D. P. Leonard , S. Maurya , E. J. Park , L. Delfin Manriquez , S. Noh , X. Wang , C. Bae , E. D. Baca , C. Fujimoto , Y. S. Kim , J. Mater. Chem. A 2020, 8, 14135.

[46] A. Carlson , P. Shapturenka , B. Eriksson , G. Lindbergh , C. Lagergren , R. W. Lindström , Electrochim. Acta 2018, 277, 151.

[47] D. Yang , H. Yu , G. Li , Y. Zhao , Y. Liu , C. Zhang , W. Song , Z. Shao , J. Power Sources 2014, 267, 39.

[48] T. J. Omasta , Y. Zhang , A. M. Park , X. Peng , B. Pivovar , J. R. Varcoe , W. E. Mustain , J. Electrochem. Soc. 2018, 165, F710.

[49] DOE Technical Targets for Polymer Electrolyte Membrane Fuel Cell Components, https://www.energy.gov/eere/fuelcells/doe‐technical‐targets‐polymer‐electrolyte‐membrane‐fuel‐cell‐components (accessed: January 2021).

[50] A. G. Wright , J. Fan , B. Britton , T. Weissbach , H.‐F. Lee , E. A. Kitching , T. J. Peckham , S. Holdcroft , Energy Environ. Sci. 2016, 9, 2130.

[51] C. Chen , J. Pan , J. Han , Y. Wang , L. Zhu , M. A. Hickner , L. Zhuang , J. Mater. Chem. A 2016, 4, 4071.

[52] J. Pan , L. Zhu , J. Han , M. A. Hickner , Chem. Mater. 2015, 27, 6689.

[53] J. Ponce‐González , D. K. Whelligan , L. Wang , R. Bance‐Soualhi , Y. Wang , Y. Peng , H. Peng , D. C. Apperley , H. N. Sarode , T. P. Pandey , A. G. Divekar , S. Seifert , A. M. Herring , L. Zhuang , J. R. Varcoe , Energy Environ. Sci. 2016, 9, 3724.

[54] M. Mandal , G. Huang , N. U. Hassan , X. Peng , T. Gu , A. H. Brooks‐Starks , B. Bahar , W. E. Mustain , P. A. Kohl , J. Electrochem. Soc. 2019, 167, 054501.

[55] S. Jang , M. Her , S. Kim , J. H. Jang , J. E. Chae , J. Choi , M. Choi , S. M. Kim , H. J. Kim , Y. H. Cho , Y. E. Sung , S. J. Yoo , ACS Appl. Mater. Interfaces 2019, 11, 34805. 31469540 10.1021/acsami.9b08075

[56] K. Ehelebe , D. Seeberger , M. T. Y. Paul , S. Thiele , K. J. J. Mayrhofer , S. Cherevko , J. Electrochem. Soc. 2019, 166, F1259.

[57] P. Mardle , G. Thirunavukkarasu , S. Guan , Y. L. Chiu , S. Du , ACS Appl. Mater. Interfaces 2020, 12, 42832. 32865384 10.1021/acsami.0c11531

[58] K. Ehelebe , T. Ashraf , S. Hager , D. Seeberger , S. Thiele , S. Cherevko , Electrochem. Commun. 2020, 116, 106761.

[59] H. Wang , H. D. Abruna , J. Am. Chem. Soc. 2017, 139, 6807. 28460520 10.1021/jacs.7b02434

[60] Q. Li , H. Peng , Y. Wang , L. Xiao , J. Lu , L. Zhuang , Angew. Chem., Int. Ed. 2019, 58, 1442. 10.1002/anie.201812662 30548378

[61] R. Wang , D. Li , S. Maurya , Y. S. Kim , Y. Wu , Y. Liu , D. Strmcnik , N. M. Markovic , V. R. Stamenkovic , Nanoscale Horiz. 2020, 5, 316.

[62] W. Xiao , W. Lei , J. Wang , G. Gao , T. Zhao , M. A. L. Cordeiro , R. Lin , M. Gong , X. Guo , E. Stavitski , H. L. Xin , Y. Zhu , D. Wang , J. Mater. Chem. A 2018, 6, 11346.

[63] M. H. Shao , K. Sasaki , R. R. Adzic , J. Am. Chem. Soc. 2006, 128, 3526. 16536519 10.1021/ja060167d

[64] H. A. Miller , A. Lavacchi , F. Vizza , M. Marelli , F. Di Benedetto , F. D'Acapito , Y. Paska , M. Page , D. R. Dekel , Angew. Chem., Int. Ed. 2016, 55, 6004. 10.1002/anie.201600647 27062251

[65] M. Bellini , M. V. Pagliaro , A. Lenarda , P. Fornasiero , M. Marelli , C. Evangelisti , M. Innocenti , Q. Jia , S. Mukerjee , J. Jankovic , L. Wang , J. R. Varcoe , C. B. Krishnamurthy , I. Grinberg , E. Davydova , D. R. Dekel , H. A. Miller , F. Vizza , ACS Appl. Energy Mater. 2019, 2, 4999.

[66] R. K. Singh , E. S. Davydova , J. Douglin , A. O. Godoy , H. Tan , M. Bellini , B. J. Allen , J. Jankovic , H. A. Miller , A. C. Alba‐Rubio , D. R. Dekel , Adv. Funct. Mater. 2020, 30, 2002087.

[67] H. Yu , E. S. Davydova , U. Ash , H. A. Miller , L. Bonville , D. R. Dekel , R. Maric , Nano Energy 2019, 57, 820.

[68] J. Ohyama , D. Kumada , A. Satsuma , J. Mater. Chem. A 2016, 4, 15980.

[69] D. Liu , S. Lu , Y. Xue , Z. Guan , J. Fang , W. Zhu , Z. Zhuang , Nano Energy 2019, 59, 26.

[70] F. Song , W. Li , J. Yang , G. Han , P. Liao , Y. Sun , Nat. Commun. 2018, 9, 4531. 30382092 10.1038/s41467-018-06728-7 PMC6208398

[71] F. Yang , X. Bao , Y. Zhao , X. Wang , G. Cheng , W. Luo , J. Mater. Chem. A 2019, 7, 10936.

[72] W. Ni , A. Krammer , C. S. Hsu , H. M. Chen , A. Schuler , X. Hu , Angew. Chem., Int. Ed. 2019, 58, 7445. 10.1002/anie.201902751 30951227

[73] S. Kabir , K. Lemire , K. Artyushkova , A. Roy , M. Odgaard , D. Schlueter , A. Oshchepkov , A. Bonnefont , E. Savinova , D. C. Sabarirajan , P. Mandal , E. J. Crumlin , I. V. Zenyuk , P. Atanassov , A. Serov , J. Mater. Chem. A 2017, 5, 24433.

[74] L. Wang , Z. Tang , W. Yan , Q. Wang , H. Yang , S. Chen , J. Power Sources 2017, 343, 458.

[75] S. Wang , L. Xiong , J. Bi , X. Zhang , G. Yang , S. Yang , ACS Appl. Mater. Interfaces 2018, 10, 27009. 30040371 10.1021/acsami.8b07742

[76] E. J. Coleman , M. H. Chowdhury , A. C. Co , ACS Catal. 2015, 5, 1245.

[77] X. Y. Huang , A. J. Wang , X. F. Zhang , L. Zhang , J. J. Feng , ACS Appl. Energy Mater. 2018, 1, 5779.

[78] N. Du , C. Wang , R. Long , Y. Xiong , Nano Res. 2017, 10, 3228.

[79] Y. Xiong , Y. Yang , F. J. DiSalvo , H. D. Abruna , ACS Nano 2020, 14, 13069. 32935972 10.1021/acsnano.0c04559

[80] Y. Wang , Y. Yang , S. Jia , X. Wang , K. Lyu , Y. Peng , H. Zheng , X. Wei , H. Ren , L. Xiao , J. Wang , D. A. Muller , H. D. Abruna , B. J. Hwang , J. Lu , L. Zhuang , Nat. Commun. 2019, 10, 1506. 30944328 10.1038/s41467-019-09503-4 PMC6447550

[81] J.‐D. Yi , R. Xu , Q. Wu , T. Zhang , K.‐T. Zang , J. Luo , Y.‐L. Liang , Y.‐B. Huang , R. Cao , ACS Energy Lett. 2018, 3, 883.

[82] H. A. Miller , M. V. Pagliaro , M. Bellini , F. Bartoli , L. Wang , I. Salam , J. R. Varcoe , F. Vizza , ACS Appl. Energy Mater. 2020, 3, 10209.

[83] I. Kruusenberg , L. Matisen , Q. Shah , A. M. Kannan , K. Tammeveski , Int. J. Hydrogen Energy 2012, 37, 4406.

[84] I. Kruusenberg , D. Ramani , S. Ratso , U. Joost , R. Saar , P. Rauwel , A. M. Kannan , K. Tammeveski , ChemElectroChem 2016, 3, 1455.

[85] P. Srathongluan , R. Kuhamaneechot , P. Sukthao , V. Vailikhit , S. Choopun , A. Tubtimtae , J. Colloid Interface Sci. 2016, 463, 222. 26524258 10.1016/j.jcis.2015.10.052

[86] R. Sibul , E. Kibena‐Põldsepp , S. Ratso , M. Kook , M. T. Sougrati , M. Käärik , M. Merisalu , J. Aruväli , P. Paiste , A. Treshchalov , J. Leis , V. Kisand , V. Sammelselg , S. Holdcroft , F. Jaouen , K. Tammeveski , ChemElectroChem 2020, 7, 1739.

[87] J. Zhang , W. Zhu , Y. Pei , Y. Liu , Y. Qin , X. Zhang , Q. Wang , Y. Yin , M. D. Guiver , ChemSusChem 2019, 12, 4165. 31368182 10.1002/cssc.201901668

[88] M. Mooste , T. Tkesheliadze , J. Kozlova , A. Kikas , V. Kisand , A. Treshchalov , A. Tamm , J. Aruväli , J. H. Zagal , A. M. Kannan , K. Tammeveski , Int. J. Hydrogen Energy 2021, 46, 4365.

[89] S. Ratso , A. Zitolo , M. Käärik , M. Merisalu , A. Kikas , V. Kisand , M. Rähn , P. Paiste , J. Leis , V. Sammelselg , S. Holdcroft , F. Jaouen , K. Tammeveski , Renewable Energy 2021, 167, 800.

[90] R. Praats , M. Käärik , A. Kikas , V. Kisand , J. Aruväli , P. Paiste , M. Merisalu , A. Sarapuu , J. Leis , V. Sammelselg , J. C. Douglin , D. R. Dekel , K. Tammeveski , J. Solid State Electrochem. 2021, 25, 57.

[91] S. Kabir , A. Serov , K. Artyushkova , P. Atanassov , ACS Catal. 2017, 7, 6609.

[92] M.‐J. Kim , J. E. Park , S. Kim , M. S. Lim , A. Jin , O.‐H. Kim , M. J. Kim , K.‐S. Lee , J. Kim , S.‐S. Kim , Y.‐H. Cho , Y.‐E. Sung , ACS Catal. 2019, 9, 3389.

[93] Y. J. Sa , C. Park , H. Y. Jeong , S. H. Park , Z. Lee , K. T. Kim , G. G. Park , S. H. Joo , Angew. Chem., Int. Ed. 2014, 53, 4102. 10.1002/anie.201307203 24554521

[94] O. H. Kim , Y. H. Cho , D. Y. Chung , M. J. Kim , J. M. Yoo , J. E. Park , H. Choe , Y. E. Sung , Sci. Rep. 2015, 5, 8376. 25728910 10.1038/srep08376 PMC4345340

[95] G. A. Ferrero , K. Preuss , A. Marinovic , A. B. Jorge , N. Mansor , D. J. Brett , A. B. Fuertes , M. Sevilla , M. M. Titirici , ACS Nano 2016, 10, 5922. 27214056 10.1021/acsnano.6b01247

[96] H.‐C. Huang , Y.‐C. Lin , S.‐T. Chang , C.‐C. Liu , K.‐C. Wang , H.‐P. Jhong , J.‐F. Lee , C.‐H. Wang , J. Mater. Chem. A 2017, 5, 19790.

[97] S. K. Singh , V. Kashyap , N. Manna , S. N. Bhange , R. Soni , R. Boukherroub , S. Szunerits , S. Kurungot , ACS Catal. 2017, 7, 6700.

[98] H. Ren , Y. Wang , Y. Yang , X. Tang , Y. Peng , H. Peng , L. Xiao , J. Lu , H. D. Abruña , L. Zhuang , ACS Catal. 2017, 7, 6485.

[99] S. Ratso , I. Kruusenberg , M. Käärik , M. Kook , L. Puust , R. Saar , J. Leis , K. Tammeveski , J. Power Sources 2018, 375, 233.

[100] M. M. Hossen , K. Artyushkova , P. Atanassov , A. Serov , J. Power Sources 2018, 375, 214. 10.1016/j.jpowsour.2017.11.039 PMC5738968 29398775

[101] M. Wang , H. Zhang , G. Thirunavukkarasu , I. Salam , J. R. Varcoe , P. Mardle , X. Li , S. Mu , S. Du , ACS Energy Lett. 2019, 4, 2104.

[102] Y. Yang , H. Peng , Y. Xiong , Q. Li , J. Lu , L. Xiao , F. J. DiSalvo , L. Zhuang , H. D. Abruña , ACS Energy Lett. 2019, 4, 1251.

[103] V. Men Truong , J. Richard Tolchard , J. Svendby , M. Manikandan , H. A. Miller , S. Sunde , H. Yang , D. R. Dekel , A. Oyarce Barnett , Energies 2020, 13, 582.

[104] S. Hanif , N. Iqbal , X. Shi , T. Noor , G. Ali , A. M. Kannan , Renewable Energy 2020, 154, 508.

[105] J. Lilloja , M. Mooste , E. Kibena‐Põldsepp , A. Sarapuu , B. Zulevi , A. Kikas , H.‐M. Piirsoo , A. Tamm , V. Kisand , S. Holdcroft , A. Serov , K. Tammeveski , J. Power Sources Adv. 2021, 8, 100052.

[106] K. Kisand , A. Sarapuu , D. Danilian , A. Kikas , V. Kisand , M. Rahn , A. Treshchalov , M. Kaarik , M. Merisalu , P. Paiste , J. Aruvali , J. Leis , V. Sammelselg , S. Holdcroft , K. Tammeveski , J. Colloid Interface Sci. 2021, 584, 263. 33069025 10.1016/j.jcis.2020.09.114

[107] J. H. Lee , J.‐h. Jang , J. Kim , S. J. Yoo , J. Ind. Eng. Chem. 2021, 10.1016/j.jiec.2021.03.004.

[108] S. Park , M. Her , H. Shin , W. Hwang , Y.‐E. Sung , ACS Appl. Energy Mater. 2021, 4, 1459.

[109] S. H. Lee , J. Kim , D. Y. Chung , J. M. Yoo , H. S. Lee , M. J. Kim , B. S. Mun , S. G. Kwon , Y. E. Sung , T. Hyeon , J. Am. Chem. Soc. 2019, 141, 2035. 30620877 10.1021/jacs.8b11129

[110] J. W. F. To , J. W. D. Ng , S. Siahrostami , A. L. Koh , Y. Lee , Z. Chen , K. D. Fong , S. Chen , J. He , W.‐G. Bae , J. Wilcox , H. Y. Jeong , K. Kim , F. Studt , J. K. Nørskov , T. F. Jaramillo , Z. Bao , Nano Res. 2016, 10, 1163.

[111] S. Lee , M. Choun , Y. Ye , J. Lee , Y. Mun , E. Kang , J. Hwang , Y. H. Lee , C. H. Shin , S. H. Moon , S. K. Kim , E. Lee , J. Lee , Angew. Chem., Int. Ed. 2015, 54, 9230. 10.1002/anie.201501590 26087961

[112] Y. Mun , M. J. Kim , S.‐A. Park , E. Lee , Y. Ye , S. Lee , Y.‐T. Kim , S. Kim , O.‐H. Kim , Y.‐H. Cho , Appl. Catal., B 2018, 222, 191.

[113] W. Zhu , Y. Pei , Y. Liu , J. Zhang , Y. Qin , Y. Yin , M. D. Guiver , ACS Appl. Mater. Interfaces 2020, 12, 32842. 32589022 10.1021/acsami.0c08829

[114] L. Zeng , T. S. Zhao , L. An , J. Mater. Chem. A 2015, 3, 1410.

[115] S. H. Lee , D. H. Lee , W. J. Lee , S. O. Kim , Adv. Funct. Mater. 2011, 21, 1338.

[116] J. Lilloja , E. Kibena‐Põldsepp , A. Sarapuu , A. Kikas , V. Kisand , M. Käärik , M. Merisalu , A. Treshchalov , J. Leis , V. Sammelselg , Q. Wei , S. Holdcroft , K. Tammeveski , Appl. Catal., B 2020, 272, 119012.

[117] J. Lilloja , E. Kibena‐Põldsepp , A. Sarapuu , M. Kodali , Y. Chen , T. Asset , M. Käärik , M. Merisalu , P. Paiste , J. Aruväli , A. Treshchalov , M. Rähn , J. Leis , V. Sammelselg , S. Holdcroft , P. Atanassov , K. Tammeveski , ACS Appl. Energy Mater. 2020, 3, 5375.

[118] R. Praats , M. Käärik , A. Kikas , V. Kisand , J. Aruväli , P. Paiste , M. Merisalu , J. Leis , V. Sammelselg , J. H. Zagal , S. Holdcroft , N. Nakashima , K. Tammeveski , Electrochim. Acta 2020, 334, 135575.

[119] S. Ratso , M. Käärik , M. Kook , P. Paiste , V. Kisand , S. Vlassov , J. Leis , K. Tammeveski , ChemElectroChem 2018, 5, 1827.

[120] J. Lilloja , E. Kibena‐Põldsepp , A. Sarapuu , J. C. Douglin , M. Käärik , J. Kozlova , P. Paiste , A. Kikas , J. Aruväli , J. Leis , V. Sammelselg , D. R. Dekel , K. Tammeveski , ACS Catal. 2021, 11, 1920. 10.1021/acscatal.0c03511 PMC8744415 35028188

[121] Y. S. Li , T. S. Zhao , Int. J. Hydrogen Energy 2012, 37, 15334.

[122] X. Shi , S. Ahmad , K. Pérez‐Salcedo , B. Escobar , H. Zheng , A. M. Kannan , Int. J. Hydrogen Energy 2019, 44, 1166.

[123] K. Miyazaki , T. Abe , K. Nishio , H. Nakanishi , Z. Ogumi , J. Power Sources 2010, 195, 6500.

[124] X. Liang , M. A. Shehzad , Y. Zhu , L. Wang , X. Ge , J. Zhang , Z. Yang , L. Wu , J. R. Varcoe , T. Xu , Chem. Mater. 2019, 31, 7812.

[125] T. J. Peckham , S. Holdcroft , Adv. Mater. 2010, 22, 4667. 20848594 10.1002/adma.201001164

[126] J. R. Varcoe , P. Atanassov , D. R. Dekel , A. M. Herring , M. A. Hickner , P. A. Kohl , A. R. Kucernak , W. E. Mustain , K. Nijmeijer , K. Scott , T. Xu , L. Zhuang , Energy Environ. Sci. 2014, 7, 3135.

[127] D. W. Shin , M. D. Guiver , Y. M. Lee , Chem. Rev. 2017, 117, 4759. 28257183 10.1021/acs.chemrev.6b00586

[128] Z. Yang , R. Guo , R. Malpass‐Evans , M. Carta , N. B. McKeown , M. D. Guiver , L. Wu , T. Xu , Angew. Chem., Int. Ed. 2016, 55, 11499. 10.1002/anie.201605916 27505421

[129] X. Ge , Y. He , M. D. Guiver , L. Wu , J. Ran , Z. Yang , T. Xu , Adv. Mater. 2016, 28, 3467. 26972938 10.1002/adma.201506199

[130] K. Zhang , M. B. McDonald , I. E. A. Genina , P. T. Hammond , Chem. Mater. 2018, 30, 6420.

[131] X. He , Y. Yang , H. Wu , G. He , Z. Xu , Y. Kong , L. Cao , B. Shi , Z. Zhang , C. Tongsh , K. Jiao , K. Zhu , Z. Jiang , Adv. Mater. 2020, 32, 2001284. 10.1002/adma.202001284 32715516

[132] P. Sun , F. Chen , W. Zhou , X. Liu , R. Ma , T. Sasaki , Mater. Horiz. 2019, 6, 2087.

[133] Z. Siroma , R. Kakitsubo , N. Fujiwara , T. Ioroi , S.‐i. Yamazaki , K. Yasuda , J. Power Sources 2009, 189, 994.

[134] D. K. Paul , A. Fraser , K. Karan , Electrochem. Commun. 2011, 13, 774.

[135] M. Mandal , ChemElectroChem 2020, 8, 36.

[136] G. Gupta , K. Scott , M. Mamlouk , Fuel Cells 2018, 18, 137.

[137] Y. S. Kim , L. Dong , M. A. Hickner , T. E. Glass , V. Webb , J. E. McGrath , Macromolecules 2003, 36, 6281.

[138] M. Adachi , T. Navessin , Z. Xie , F. H. Li , S. Tanaka , S. Holdcroft , J. Membr. Sci. 2010, 364, 183.

[139] S. D. Poynton , R. C. T. Slade , T. J. Omasta , W. E. Mustain , R. Escudero‐Cid , P. Ocón , J. R. Varcoe , J. Mater. Chem. A 2014, 2, 5124.

[140] Y. Zheng , U. Ash , R. P. Pandey , A. G. Ozioko , J. Ponce‐González , M. Handl , T. Weissbach , J. R. Varcoe , S. Holdcroft , M. W. Liberatore , R. Hiesgen , D. R. Dekel , Macromolecules 2018, 51, 3264.

[141] X. Chu , Y. Shi , L. Liu , Y. Huang , N. Li , J. Mater. Chem. A 2019, 7, 7717.

[142] R. Gutru , Z. Turtayeva , F. Xu , G. Maranzana , B. Vigolo , A. Desforges , Int. J. Hydrogen Energy 2020, 45, 19642.

[143] J. Choi , M.‐H. Kim , J. Y. Han , J. E. Chae , W. H. Lee , Y. M. Lee , S. Y. Lee , J. H. Jang , J. Y. Kim , D. Henkensmeier , S. J. Yoo , Y.‐E. Sung , H.‐J. Kim , J. Membr. Sci. 2018, 568, 67.

[144] M. Mukaddam , E. Litwiller , I. Pinnau , Macromolecules 2015, 49, 280.

[145] A. Z. Weber , A. Kusoglu , J. Mater. Chem. A 2014, 2, 17207.

[146] W. You , K. J. T. Noonan , G. W. Coates , Prog. Polym. Sci. 2020, 100, 101177.

[147] Y. Yin , R. Li , F. Bai , W. Zhu , Y. Qin , Y. Chang , J. Zhang , M. D. Guiver , Electrochem. Commun. 2019, 109, 106590.

[148] Y. Chang , J. Liu , R. Li , J. Zhao , Y. Qin , J. Zhang , Y. Yin , X. Li , Energy Convers. Manage. 2019, 189, 24.

[149] D. R. Dekel , M. Amar , S. Willdorf , M. Kosa , S. Dhara , C. E. Diesendruck , Chem. Mater. 2017, 29, 4425.

[150] C. G. Arges , V. Ramani , Proc. Natl. Acad. Sci. USA 2013, 110, 2490. 23335629 10.1073/pnas.1217215110 PMC3574919

[151] D. R. Dekel , J. Power Sources 2018, 375, 158.

[152] A. L. Gonçalves Biancolli , D. Herranz , L. Wang , G. Stehlíková , R. Bance‐Soualhi , J. Ponce‐González , P. Ocón , E. A. Ticianelli , D. K. Whelligan , J. R. Varcoe , E. I. Santiago , J. Mater. Chem. A 2018, 6, 24330.

[153] J. R. Varcoe , R. C. Slade , E. L. H. Yee , Chem. Commun. 2006, 1428. 10.1039/b600838k 16550289

[154] R. Espiritu , M. Mamlouk , K. Scott , Int. J. Hydrogen Energy 2016, 41, 1120.

[155] Z. Wang , J. Parrondo , V. Ramani , J. Electrochem. Soc. 2017, 164, F1216.

[156] J. S. Olsson , T. H. Pham , P. Jannasch , Adv. Funct. Mater. 2018, 28, 1702758.

[157] J. Wang , Y. Zhao , B. P. Setzler , S. Rojas‐Carbonell , C. Ben Yehuda , A. Amel , M. Page , L. Wang , K. Hu , L. Shi , S. Gottesfeld , B. Xu , Y. Yan , Nat. Energy 2019, 4, 392.

[158] H. Peng , Q. Li , M. Hu , L. Xiao , J. Lu , L. Zhuang , J. Power Sources 2018, 390, 165.

[159] S. Maurya , S. Noh , I. Matanovic , E. J. Park , C. Narvaez Villarrubia , U. Martinez , J. Han , C. Bae , Y. S. Kim , Energy Environ. Sci. 2018, 11, 3283.

[160] S. Holdcroft , Chem. Mater. 2013, 26, 381.

[161] K. More , R. Borup , K. Reeves , ECS Trans. 2006, 3, 717.

[162] M. Ünlü , D. Abbott , N. Ramaswamy , X. Ren , S. Mukerjee , P. A. Kohl , J. Electrochem. Soc. 2011, 158, B1423.

[163] S.‐D. Yim , H. T. Chung , J. Chlistunoff , D.‐S. Kim , C. Fujimoto , T.‐H. Yang , Y. S. Kim , J. Electrochem. Soc. 2015, 162, F499.

[164] H. T. Chung , Y.‐K. Choe , U. Martinez , J. H. Dumont , A. Mohanty , C. Bae , I. Matanovic , Y. S. Kim , J. Electrochem. Soc. 2016, 163, F1503.

[165] H. T. Chung , U. Martinez , I. Matanovic , Y. S. Kim , J. Phys. Chem. Lett. 2016, 7, 4464. 27771955 10.1021/acs.jpclett.6b02025

[166] S. Maurya , C. H. Fujimoto , M. R. Hibbs , C. Narvaez Villarrubia , Y. S. Kim , Chem. Mater. 2018, 30, 2188.

[167] I. Matanovic , S. Maurya , E. J. Park , J. Y. Jeon , C. Bae , Y. S. Kim , Chem. Mater. 2019, 31, 4195.

[168] S. Maurya , J. H. Dumont , C. N. Villarrubia , I. Matanovic , D. Li , Y. S. Kim , S. Noh , J. Han , C. Bae , H. A. Miller , C. H. Fujimoto , D. R. Dekel , ACS Catal. 2018, 8, 9429.

[169] Y. Kurihara , T. Mabuchi , T. Tokumasu , J. Electrochem. Soc. 2017, 164, F628.

[170] L. Fan , Y. Wang , K. Jiao , ACS Nano 2020, 14, 17487. 10.1021/acsnano.0c07856 33306905

[171] G. F. Brunello , J. H. Lee , S. G. Lee , J. I. Choi , D. Harvey , S. S. Jang , RSC Adv. 2016, 6, 69670.

[172] K. Artyushkova , M. J. Workman , I. Matanovic , M. J. Dzara , C. Ngo , S. Pylypenko , A. Serov , P. Atanassov , ACS Appl. Energy Mater. 2018, 1, 68.

[173] Z. Yang , Y. Liu , R. Guo , J. Hou , L. Wu , T. Xu , Chem. Commun. 2016, 52, 2788. 10.1039/c5cc09024e 26765494

[174] R. Ren , X. Wang , H. Chen , H. A. Miller , I. Salam , J. R. Varcoe , L. Wu , Y. Chen , H. G. Liao , E. Liu , F. Bartoli , F. Vizza , Q. Jia , Q. He , Angew. Chem., Int. Ed. 2021, 60, 4049. 10.1002/anie.202012547 33188558

